# Phytochemical Profile, Antioxidant Capacity and Anticancer Potential of Water Extracts from In Vitro Cultivated *Salvia aethiopis*

**DOI:** 10.3390/molecules30071427

**Published:** 2025-03-23

**Authors:** Krasimira Tasheva, Inna Sulikovska, Ani Georgieva, Vera Djeliova, Vesela Lozanova, Anelia Vasileva, Ivaylo Ivanov, Petko Denev, Maria Lazarova, Valya Vassileva, Polina Petkova-Kirova

**Affiliations:** 1 Institute of Plant Physiology and Genetics, Bulgarian Academy of Sciences, Acad. G. Bonchev Str., Bl. 21, 1113 Sofia, Bulgaria; krasitasheva@abv.bg; 2 Department of Pathology, Institute of Experimental Morphology, Pathology and Anthropology with Museum, Bulgarian Academy of Sciences, 1113 Sofia, Bulgaria; inna_sulikovska@ukr.net (I.S.); georgieva_any@abv.bg (A.G.); 3 Department of Molecular Biology of Cell Cycle, Institute of Molecular Biology “Acad. R. Tsanev”, Bulgarian Academy of Sciences, Acad. G. Bonchev Str., Bl. 21, 1113 Sofia, Bulgaria; vera@bio21.bas.bg; 4 Department of Medical Chemistry and Biochemistry, Medical University–Sofia, 1431 Sofia, Bulgaria; vlozanova@medfac.mu-sofia.bg (V.L.); avasileva@medfac.mu-sofia.bg (A.V.); iivanov@medfac.mu-sofia.bg (I.I.); 5 Laboratory of Biologically Active Substances, Institute of Organic Chemistry with Centre of Phytochemistry, Bulgarian Academy of Sciences, 4000 Plovdiv, Bulgaria; petko.denev@orgchm.bas.bg; 6 Department of Synaptic Signaling and Communication, Institute of Neurobiology, Bulgarian Academy of Sciences, 1113 Sofia, Bulgaria; m.lazarova@gmail.com (M.L.); kirovaps@yahoo.com (P.P.-K.)

**Keywords:** *Salvia aethiopis*, phytochemicals, antioxidant activity, hepatocellular carcinoma, cytotoxicity, apoptosis, LC-HRMS

## Abstract

*Salvia aethiopis* L. (Mediterranean sage) is a medicinal plant known for its rich phenolic content and different therapeutic properties. This study evaluated the phytochemical composition, antioxidant capacity and anticancer potential of water extracts from in vitro cultivated *S. aethiopis*. The extract exhibited a high total polyphenol (110.03 ± 0.7 mg GAE/g) and flavonoid (7.88 ± 0.25 mg QE/g) content, along with a strong oxygen radical absorbance capacity (an ORAC value of 3677.9 ± 24.8 µmol TE/g). LC-HRMS analysis identified 21 bioactive compounds, including salvianic acid C, rosmarinic acid, salvianolic acid K and various organic acids. A cytotoxicity evaluation using the Neutral Red Uptake assay showed that the extract had a low toxicity to non-cancerous BALB/3T3 cells. An antiproliferative activity assessment via the MTT assay revealed selective cytotoxicity against Hep G2 hepatocellular carcinoma cells (IC_50_ = 353.8 ± 21.8 µg/mL) and lung (A549) and prostate (PC-3) carcinoma cell lines. Migration assays and cytopathological evaluations confirmed the significant inhibition of cancer cell proliferation, the suppression of migration and G2/M cell cycle arrest. Flow cytometry revealed considerable increases in apoptotic and necrotic cell populations following treatment with *S. aethiopis* extract. These findings showed the potential of *S. aethiopis* as a promising source of bioactive compounds with antioxidant and anticancer properties, supporting its further exploration for therapeutic applications.

## 1. Introduction

Aromatic and medicinal plants release a number of secondary metabolites like flavonoids and other polyphenols with a wide range of beneficial effects. Their potent antioxidant properties through scavenging reactive oxygen species (ROS), the activation of antioxidant enzymes, the inhibition of oxidases and the reduction of α-tocopheryl radicals [1,2] are complemented by their anti-inflammatory, antimicrobial, anti-aging, anticancer, cardioprotective, neuroprotective and UV-protective activities [3]. The multifunctional nature of these compounds underpins their importance not only in traditional medicine but also in the modern development of pharmaceutical products with preventive and therapeutic applications [4].

Plants of the genus *Salvia* (Lamiaceae family) have long been recognized for their medicinal properties and widely used in traditional medicine [5]. Among them, *Salvia aethiopis* L. (Mediterranean or African sage) synthesizes several types of valuable secondary metabolites, amongst which are abietane diterpenoids like aethiopinone salvipisone and sesterpenes [6,7,8]. It also produces phenolic acids like rosmarinic and caffeic acid [5,9,10], which contribute to its broad spectrum of bioactivities—antimicrobial, antifungal, antimycobacterial, antioxidant, anti-inflammatory and anti-cholinesterase effects [3,11,12,13,14,15,16,17]. Notably, *S. aethiopis* also exhibits cytotoxic properties against different cancer cell lines, with ethiopinone, for example, shown to induce apoptosis in human melanoma cells [18]. However, available data on the anticancer activity of *S. aethiopis* L. remain scarce and limited to extracts obtained using organic solvents [10,14,19]. To our knowledge, the anticancer potential of in vitro obtained and cultivated *S. aethiopis* plants has not yet been investigated.

Cancer, often referred to as the disease of modern life, remains one of the leading causes of premature death worldwide [20]. Due to global population growth and aging, the cancer burden is projected to rise to 21.4 million new cases and 13.2 million deaths annually by 2030 [21,22]. Despite extensive research, the precise etiology of many neoplastic diseases remains unclear. However, oxidative stress, oxidative DNA damage and chronic inflammation have been identified as specific risk factors [23,24]. Currently, cancer is not curable, and conventional treatment methods such as surgery, radiation therapy and chemotherapy often cause damage to healthy cells and tissues. Given their rich composition of bioactive secondary metabolites, medicinal plants with a demonstrated cytotoxic activity against cancer cells represent a promising avenue for the development of safer, more affordable and effective anticancer therapies.

This research aimed to explore the chemical composition, antioxidant capacity and anticancer effects of water extracts from cultivated *S. aethiopis,* as neither in vitro obtained and cultivated plants nor the water extracts of those have been studied so far. The study examined the antiproliferative activity of the *S. aethiopis* extract on A540 lung, PC-3 prostate and Hep G2 hepatocellular carcinoma cell lines (used as in vitro models for some of the most common cancer types in humans), for which no data on the effects of *S. aethiopis* extracts is available in the literature. The effects of the extract on cell migration, cell cycle progression and apoptosis were also examined to determine whether the anticipated anticancer activity of *S. aethiopis* was supported by antimetastatic properties, cancer cell cycle arrest and pathological alterations in the cancer cells.

## 2. Results

### 2.1. Phytochemical Analyses

#### 2.1.1. Total Polyphenol and Flavonoid Content and Antioxidant Activity

The total polyphenol and flavonoid content, along with the antioxidant activity of freeze-dried extracts from in vitro cultivated *S. aethiopis* plants, are presented in Table 1. The extracts contained 110.03 ± 0.7 µg GAE/g of total polyphenols and 7.88 ± 0.25 µg QE/g of total flavonoids. The antioxidant activity, assessed by the ORAC assay, was 3677.9 ± 24.8 µmol TE/g, and by the HORAC assay it was 889.6 ± 14.3 µmol GAE/g, indicating a strong capacity for scavenging free radicals.

#### 2.1.2. LC-HRMS Analysis

An LC-HRMS analysis in negative ionization mode was performed to identify the phytochemical compounds of the investigated *S. aethiopis* L. extract. Compounds were identified based on MS and MS^2^ data analysis, comparing their retention times and fragmentation patterns with standard compounds and previously reported data. The results are summarized in Table 2.

The dominant constituent (compound **12** in Table 2) (Figures S1 and S2 in the Supplementary Material), exhibited a molecular ion [M-H]^−^ at *m*/*z* 359.0722, corresponding to the molecular formula C_18_H_16_O_8_. The MS^2^ spectrum revealed high-intensity fragment ions at *m*/*z* 161, 197 and 179, which are characteristic of rosmarinic acid fragmentation, rosmarinic acid being a major polyphenol compound in the Lamiaceae family. Another dominant compound identified was salvianic acid C (compound **9**) with an [M-H]^−^ ion at *m*/*z* 377.0832, corresponding to the molecular formula C_18_H_18_O_9_. Analysis of the MS/MS spectrum of compound **10** (an [M-H]^−^ ion at *m*/*z* 569.1068 and molecular formula C_24_H_26_O_16_) suggested that the compound was an ester of salvianic acid C and 2,3,4,5-tetrahydroxyhexanedioic acid, supported by the observed in the MS^2^ spectrum fragment ions at *m*/*z* 389 ([M-H-180]^−^), 371 ([M-H-180-H_2_O]^−^), 327 ([M-H-180-H_2_O-CO_2_]^−^), 197, 153 and 129 ([209-2H_2_O-CO_2_]^−^) being some of the highest peaks. An ion [M-H]^−^ at *m*/*z* 341.1038 (C_12_H_22_O_11_), exhibited fragment peaks at *m*/*z* 161 ([M-H-C_6_H_12_O_6_]), corresponding to a neutral loss of hexose, and at *m*/*z* 179, corresponding to a neutral loss of a hexose moiety, suggesting the compound to be a disaccharide composed of two hexoses (compound **2**). Compound **3** with the molecular formula C_4_H_6_O_5_, was identified as malic acid [25]. The MS/MS spectrum of compound **4**, with an [M-H]^−^ ion at *m*/*z* 191.0172 (C_6_H_8_O_7_), exhibited a significant peak at *m*/*z* 111 [M-H-CO_2_-2H_2_O]. Thus, compound **4** was deduced as citric acid [25].

The analysis of compound **13** (*m*/*z* 475.0817, molecular formula C_22_H_20_O_12_), indicating the presence of different fragment ions at *m*/*z* 299 ([M-H-176]^−^) after the loss of a glucuronic acid moiety, 284 [M-H-176-CH_3_]^−^, 175 a [glucuronic acid moiety]^−^ and 113, identified it as a methoxylated flavonoid-*O*-glucuronide. In the MS/MS spectra of four other compounds (**11**, **16**, **18** and **19**) an ion at *m*/*z* 113 was observed. In the MS/MS spectrum of compound **11** (an [M-H]^−^ at *m*/*z* 651.1111), the ion fragment at *m*/*z* 351, corresponding to an *O*-glucuronyl-glucuronic acid moiety, was the most significant, identifying compound **11** as an *O*-(*O*-glucuronyl-*O*-glucuronide)methoxylated flavonoid. Its MS/MS spectrum showed fragment ions also at *m*/*z* 299 [a methoxylated flavonoid]^−^ and 193 [glucuronic acid]^-^, confirming compound **11** as an *O*-(*O*-glucuronyl-*O*-glucuronide)methoxylated flavonoid. In the MS/MS spectrum of compound **16** (an [M-H]^−^ at *m*/*z* 813.1404 and molecular formula C_37_H_34_O_21_), fragment ions at *m*/*z* 633 ([M-H-C_9_H_8_O_4_]^−^) after a neutral loss of caffeic acid, 513 ([M-H-C_16_H_12_O_6_]^−^) after a neutral loss of a methoxylated flavonoid, 351 corresponding to an [*O*-glucuronyl-glucuronic acid moiety]^−^ and 337 corresponding to an [*O*-caffeoyl-glucuronic acid moiety]^−^, were identified. This compound we therefore considered to be an *O*-(*O*-caffeoyl-*O*-glucuronyl-*O*-glucuronide)methoxylated flavonoid. Compounds **18** (an [M-H]^−^ ion at *m*/*z* 857.1658) and **19** (an [M-H]^−^ ion at *m*/*z* 827.1561) showed fragmentation patterns similar to that of compound **16**. These compounds were considered to be an *O*-(*O*-sinapoyl-*O*-glucuronyl-*O*-glucuronide)methoxylated flavonoid and an *O*-(*O*-feruloyl-*O*-glucuronyl-*O*-glucuronide)methoxylated flavonoid, respectively. Such types of compounds, according to the information from the Lotus natural products database, are isolated from *Medicago sativa* L. [31].

The [M-H]^−^ ion at *m*/*z* 475.1754 of compound **8** corresponded to the molecular formula C_21_H_32_O_12_. The neutral loss of 146 Da (a rhamnosyl moiety) leading to the formation of an ion at *m*/*z* 329, the ion at *m*/*z* 161 corresponding to a [glucose moiety]^−^, the ion at *m*/*z* 149 corresponding to a [5-ethenyl-2-methoxyphenol]^−^ and the ion with highest relative abundance observed in the MS/MS spectrum at *m*/*z* 113 ([161-CH_2_O-H_2_O]) identified compound **8** as a 2-(3-hydroxy-4-methoxyphenyl)ethyl-*O*-(rhamnosyl)glucupyranoside. In its MS spectrum, compound **21** showed an [M-H]^−^ ion at *m*/*z* 313.0675 (C_17_H_14_O_6_). The fragment ion at *m*/*z* 161 as a base peak was associated with a [caffeic acid moiety]^−^ and the fragment ion at *m*/*z* 151 corresponded to a [(3,4-dihydroxyphenyl)acetaldehyde]^−^ suggesting that compound **21** was a 2-(3,4-dihydroxyphenyl)ethenyl 3-(3,4-dihydroxyphenyl)prop-2-enoate. Compound **17**, with an [M-H]^−^ ion at *m*/*z* 555.1065 based on data in the literature as shown in Table 2, was found to be salvianolic acid K. Two other compounds with [M-H]^−^ ions at *m*/*z* 207.0634 and 629.2346 were found in the extract but have not been identified.

According to the data in the literature, compounds **3**, **5**, **13** and **20** were found in extracts of *Salvia aethiopis* [10], and compounds **2** [25,27], **17** [27], **6**, **7** and **13** [25] and compound **21** [32] were discovered in extracts of other representatives of *Salvia* species. It should be noted that according to the already published scientific data, the twelve compounds (**1**, **4**, **8**–**10**, **11**, **14**–**16**, **18** and **19**), have not been identified so far in *S. aethiopis* extracts. The MS/MS spectra of all the compounds in Table 2 are presented in Figures S3–S23. The molecular structures of some of the dominant compounds of the extract of *S. aethiopis* are presented in Figure S24.

In summary, the presented results reveal the rich phytochemical composition of *S. aethiopis* aqueous extracts, with the major constituents being mostly rosmarinic acid and salvianic acid C but also danshensu, malic acid, isopropylmalic acid, gluconic acid and citric acid. Of note, also, is the presence of salvianolic acid K as a representative of a major class of phenolic acids–salvianolic acids, with important bioactive properties.

### 2.2. Assessment of Cytotoxicity and Antiproliferative Activity of S. aetiopis Extract

#### 2.2.1. Cytotoxicity Evaluation

**Figure 1:**
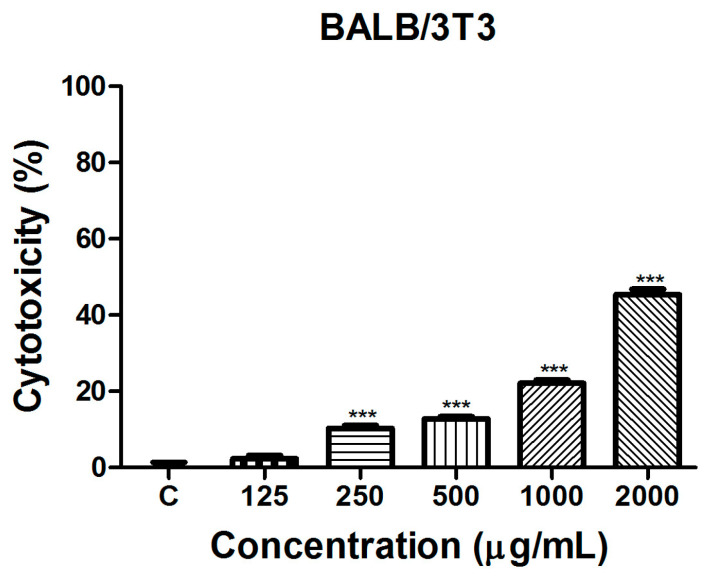
Cytotoxicity of *S. aethiopis* extract on BALB/3T3 cells assessed by the Neutral Red Uptake (NRU) test. Data are expressed as mean ± SD. Statistical significance: *** *p* < 0.001.

The in vitro safety assessment of the *S. aethiopis* extract was conducted using the standard BALB/3T3 Neutral Red Uptake (NRU) cytotoxicity assay. The test evaluated the cell viability and potential cytotoxic effects of the extract. The results of the cytotoxicity evaluation are presented in Figure 1.

As evident from the presented data, the tested extract showed a very low cytotoxicity. At concentrations of 250 µg/mL, 500 µg/mL and 1000 µg/mL, cell viability was reduced by 10.30 ± 1.89%, 12.81 ± 1.597% and 22.16 ± 2.160%, respectively. Even at the highest tested concentration of 2000 µg/mL, cytotoxicity remained below 50%, demonstrating a relatively low toxic effect on BALB/3T3 cells.

#### 2.2.2. Antiproliferative Activity

**Figure 2:**
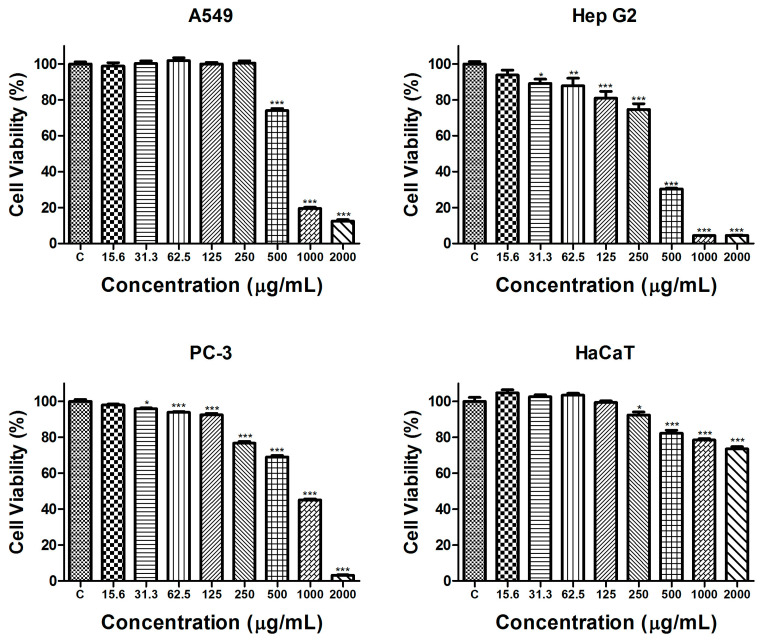
Antiproliferative effect of *S. aethiopis* extract on the cell viability of human cell lines assessed by the MTT test after 72 h of exposure. Data are presented as means ± SD. Statistical significance: * *p* < 0.05, ** *p* < 0.01 and *** *p* < 0.001.

The potential of *S. aethiopis* extract to inhibit the proliferation of cancer cells was evaluated by the MTT (3-(4,5-dimethylthiazol-2-yl)-2,5-diphenyltetrazolium bromide) assay following 72 h of exposure. To assess the selectivity of its antiproliferative effect, the effect of the extract on cancer cells was compared to its effect on the human non-cancerous HaCaT cell line. The results of the assay are presented in Figure 2.

A concentration-dependent antiproliferative effect of the tested extract with varying degrees of sensitivity in different cell lines was detected. In lung carcinoma cells (A549), a statistically significant decrease in cell proliferation was found only at the highest concentrations of 500 µg/mL, 1000 µg/mL and 2000 µg/mL. In contrast, hepatocellular and prostate carcinoma cells showed greater sensitivity, with significant inhibition occurring at all concentrations above 30 µg/mL. In the non-cancerous HaCaT cell line, the effects of the extract on cell proliferation were significantly weaker. Even at the highest tested concentration, cell viability remained at 73.6 ± 2.87% compared to untreated control cells. To further evaluate the antiproliferative potential of the *S. aethiopis* extract in different cancer cell lines, the half-maximal inhibitory concentration (IC_50_) and selectivity index (SI), relative to the non-cancerous control cell line, were determined (Table 3).

The results indicate that the extract showed stronger effects and the highest selectivity in the hepatocellular carcinoma (Hep G2) cell line. Based on the MTT assay findings, Hep G2 was identified as the most suitable model for further studies on the mechanisms underlying the observed antiproliferative effects.

#### 2.2.3. Migration Assay

**Figure 3:**
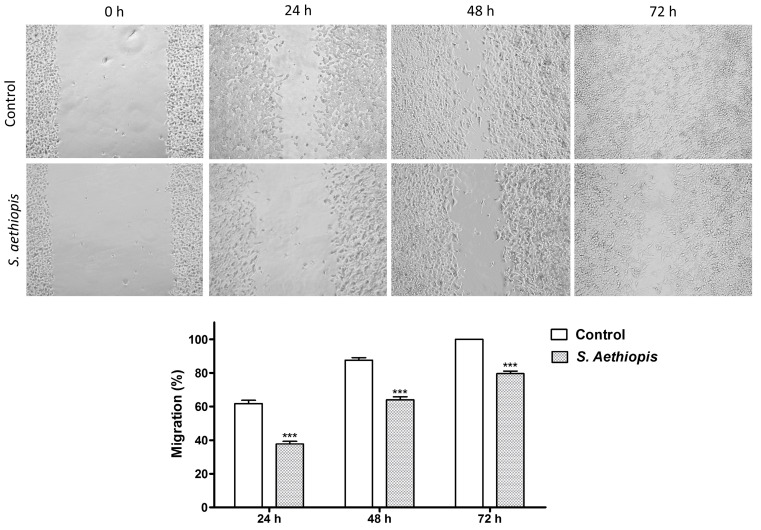
Effect of *S. aethiopis* extract on the migration capacity of hepatocellular carcinoma (Hep G2) cells. Statistical significance: *** *p* < 0.001.

The effect of *S. aethiopis* extract on cancer cell migration was evaluated using the wound-healing scratch assay (Figure 3). Cell migration was quantified by measuring the mean wound area at different time points and comparing it to the initial (0 h) scratch area in the corresponding cell culture.

In the control cell cultures, 61.72%, 87.54% and 100.00% of the wounded area was healed at 24 h, 48 h and 72 h, respectively. Treatment with *S. aethiopis* extract significantly inhibited the migration of Hep G2 carcinoma cells, reducing wound closure to 37.70%, 63.94% and 79.55% at the corresponding time points.

#### 2.2.4. Cytopathological Analysis

**Figure 4:**
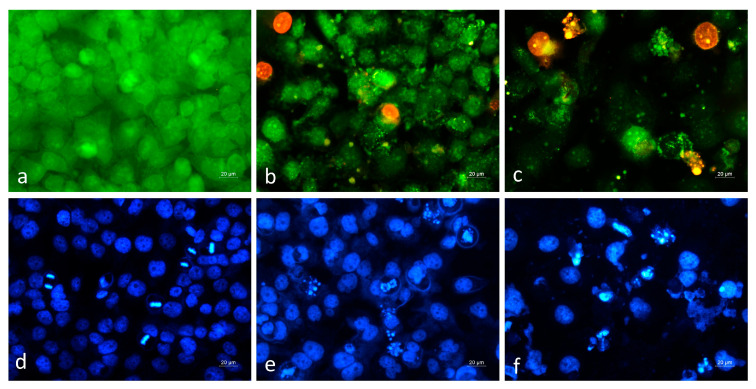
Cytopathological alterations induced by *S. aethiopis* extract in hepatocellular carcinoma (Hep G2) cells. (**a**,**d**) Negative control (untreated cells); (**b**,**e**) cells treated with *S. aethiopis* extract (300 µg/mL); (**c**,**f**) positive control (cells treated with 6 µg/mL doxorubicin). Upper panel: AO/EB staining. Lower panel: DAPI staining. Fluorescence microscopy, 40× objective.

Cytopathological changes in Hep G2 cancer cells following treatment with *S. aethiopis* extract were compared to those in cell cultures treated with the standard cytostatic agent, doxorubicin (Figure 4).

Fluorescence microscopy revealed that in the control untreated cell cultures stained with AO/EB, a monolayer of green-stained viable cells was observed, with numerous cells actively undergoing mitosis (Figure 4a). Treatment with *S. aethiopis* extract led to pronounced morphological alterations, including a reduction in monolayer confluency and the presence of early apoptotic, late apoptotic and necrotic cells (Figure 4b). Cells treated with the positive control, doxorubicin, showed similar morphological changes; however, a higher number of cells displayed typical apoptotic features, such as nuclear condensation and fragmentation (Figure 4c). Further confirmation of the ability of *S. aethiopis* extract to induce cancer cell death via apoptotic and necrotic pathways was provided by DAPI nuclear staining. Cells treated with *S. aethiopis* extract showed characteristic apoptotic nuclear alterations, though these changes were more frequent and pronounced in doxorubicin-treated cultures (Figure 4e,f).

#### 2.2.5. Cell Cycle Analysis

**Figure 5:**
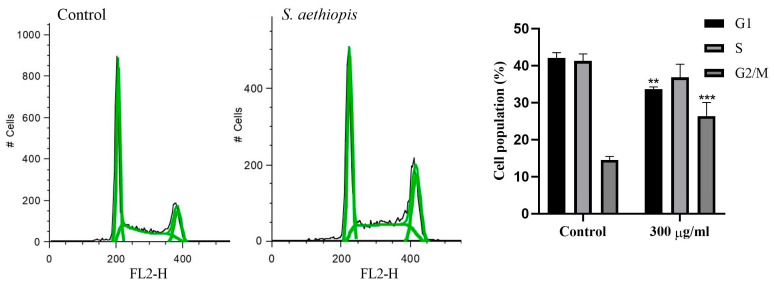
Effect of *S. aethiopis* extract on the cell cycle progression of Hep G2 hepatocellular carcinoma cells. Cells were analyzed for distribution in the individual phases of the cell cycle by examining FL2 peak heights (FL2-H, Fluorescence Channel 2—Height) (left and middle panel). Statistical significance: ** *p* < 0.01 and *** *p* < 0.001.

The effect of *S. aethiopis* extract on cell cycle progression was assessed by a fluorescence-activated cell sorting (FACS) analysis. Propidium iodide (PI)-stained control and extract-treated Hep G2 cells were examined for changes in cell cycle distribution (Figure 5). The cells were analyzed for distribution in the individual phases of the cell cycle by assessing FL2 peak heights (FL2-H, Fluorescence Channel 2—Height).

As evident from the data presented in Figure 5, exposure to *S. aethiopis* extract led to significant changes in cell cycle phase distribution. Compared to untreated control cells, the extract caused a decrease in G1- and S-phase cell populations, while the G2/M-phase population showed a statistically significant increase.

#### 2.2.6. Apoptosis Assay

**Figure 6:**
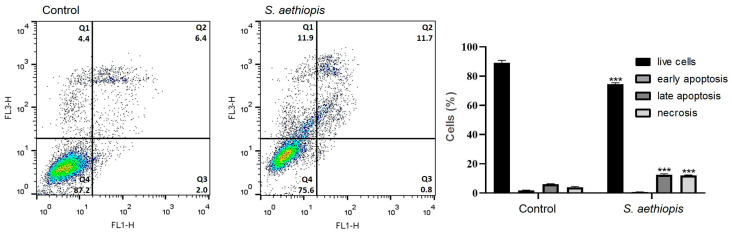
Apoptotic response of Hep G2 hepatocellular carcinoma cells following treatment with *S. aethiopis* extract as assessed by flow cytometry. Statistical significance: *** *p* < 0.001.

A flow cytometry analysis of Annexin V-FITC/PI-stained Hep G2 cells was performed to quantify the live, apoptotic and necrotic cell populations in untreated and extract-treated cell cultures (Figure 6).

The results of the Annexin V-FITC/PI FACS analysis were consistent with the data obtained from fluorescent microscopy and showed a statistically significant increase in both late apoptotic and necrotic cell populations following treatment with *S. aethiopis* extract.

## 3. Discussion

In the present research, the anticancer potential of water extracts from cultivated *S. aethiopis* plants on lung carcinoma A549, prostate adenocarcinoma PC-3 and hepatocellular carcinoma Hep G2 cell lines, used as in vitro models for some of the most common cancer types in humans, was investigated. The chemical composition and antioxidant properties of the *S. aethiopis* extract were also analyzed.

In a first step, the safety of the *S. aethiopis* extract was evaluated using the standard BALB/3T3 Neutral Red Uptake (NRU) cytotoxicity test. This test is a validated in vitro method for the assessment of basal cytotoxicity, with high predictive value for human acute toxicity. The assay measures cell viability by assessing the retention of a neutral red dye in lysosomes, distinguishing viable from non-viable cells. Neutral red (3-amino-7-dimethylamino-2-methylphenazine hydrochloride) is a weak cationic dye that readily diffuses through the cell membrane and accumulates in the lysosomes of viable cells, whereas it is excluded from dead cells. The NRU test results indicated the very low cytotoxicity of the extract, supporting its potential for therapeutic uses.

Next, the antiproliferative potential of the *S. aethiopis* extract was assessed on human A549 (lung carcinoma), PC-3 (prostate adenocarcinoma) and Hep G2 (hepatocellular carcinoma) cancer cells using the MTT assay. To evaluate the impact of the *S. aethiopis* extract on cell proliferation, a low seeding density (1000 cells/well) and long exposure time (72 h) were used. The non-cancer human cell line HaCaT, treated under the same conditions, served as a control to assess selectivity. The results showed that the tested extract inhibited the proliferation of all cell lines in a concentration-dependent manner. Among the cancer cell lines, Hep G2 exhibited the highest sensitivity to the extract, followed by A549 and PC-3, whereas HaCaT cells were the least affected, showing once again the safety of the extract on normal cells, a significant advantage when developing new anticancer treatments. Selectivity was quantified using the selectivity index (SI), calculated as the IC_50_ ratio of cancer versus normal cells. The SI is an important parameter for evaluating the anticancer effect of a compound, as it indicates the compound’s ability to preferentially target cancer cells while sparing normal, healthy cells. The obtained data indicated that the *S. aethiopis* extract demonstrated high selectivity towards the Hep G2 cells and moderate selectivity against the A549 and PC-3 cells.

Existing literature on the anticancer activity of *S. aethiopis* L. is scarce and limited to extracts obtained with organic solvents [10,19]. Additionally, no studies have investigated the anticancer potential of in vitro cultivated *S. aethiopis*, nor has its activity been evaluated against liver, lung and prostate cancer. Thus, Firuzi et al. [19] studied the cytotoxicity of methanol and dichloromethane extracts of eleven *Salvia* species, including *S. aethiopis*, against HL60 (human acute promyelocytic leukemia cells), K562 (human chronic myelogenous leukemia cells) and MCF-7 (human breast adenocarcinoma) and reported the significant cytotoxic activity of S. aethiopis against all the tested cell lines. Luca et al. [10] also conducted a study on the potential activity of S. aethiopis against breast cancer. They investigated the cytotoxic effects of ethanol extracts from S. aethiopis on human breast carcinoma MCF-7 and MDA-MB-231 (triple-negative breast cancer), but their findings indicated no significant reduction in the cell viability over the concentration range tested (25–100 μg/mL).

To our knowledge, the present study is the first to investigate the anticancer effects of water extracts from in vitro cultivated *S. aethiopis* on liver, lung and prostate cancer, demonstrating significant cytotoxic activity against all three cancer types, with the strongest impact observed on Hep G2 liver cancer cells.

To elucidate the mechanisms underlying the antiproliferative activity of *S. aethiopis* extracts, cell migration, cytomorphological and flow cytometric analyses were performed using Hep G2 cells, selected for their high responsiveness. The wound-healing (scratch) assay demonstrated the ability of the tested extract to inhibit cancer cell migration, consistent with previous studies reporting that different extracts from *Salvia* spp. and their bioactive compounds suppress cancer cell migration and metastasis in in vitro and in vivo cancer models [33,34,35]. In addition to its inhibitory effect on cancer cell migration, the *S. aethiopis* water extract was also found to suppress cancer cell proliferation by inducing apoptosis, necrosis and cell cycle arrest. The extract’s ability to disrupt cell cycle progression, evidenced by G2/M-phase arrest, suggests interference with key regulatory pathways involved in cancer cell proliferation. Similar findings, characterized by a reduction in the number of G0/G1-phase cells and the accumulation of cells in G2/M, have been reported for SW480 and HT-29 colon cancer cells treated with *Salvia rosmarinus* extracts [26,36], suggesting common bioactive compounds and anticancer mechanisms in the two *Salvia* species. The fluorescent microscopy of AO/EB- and DAPI-stained cancer cells revealed distinct apoptotic and necrotic morphological changes following *S. aethiopis* extract treatment. These effects were further confirmed and quantified by the fluorescence-activated cell sorting analysis of Annexin V-FITC/PI-stained Hep G2 cells, which demonstrated a significant increase in both late apoptotic and necrotic cell populations. Taken together with previous reports, the present findings indicate that *Salvia* spp. extracts trigger cancer cell death through different mechanisms, including both necrosis and apoptosis [34,35,36,37,38].

The strong antiproliferative and antimetastatic activity of *S. aethiopis* may be attributed to its rich phenolic content and convincing antioxidant properties. Polyphenols, particularly flavonoids, are well known for their antioxidant effects and potential role in cancer prevention and treatment [39,40].

The current analysis revealed a total polyphenol content of 110.03 ± 0.7 mg/g in the freeze-dried extract, comparable to values reported by other studies, including Tosun et al. [12] (82.1 mg GAE/g); Luca et al. [10] (81.43 mg/g); Hanganu et al. [41] (22.25 ± 0.24 mg/g); Dogan [42] (41.50 mg GAE/g); Caylak [43] (90.75 ± 0.35 mg GAE/g), and Firuzi et al. [19] (14.13 ± 0.90 mg GAE/g dried plant). Additionally, Poyraz et al. [3] reported polyphenol levels of 94.36 mg GAE/g in methanol extracts and 290.62 mg GAE/g in ethyl acetate extracts of *S. aethiopis*. However, it is important to note that, to our knowledge, only two studies examine the polyphenol and flavonoid content in extracts from cultivated *S. aethiopis* plants [10,12], and no reports exist for in vitro obtained and further cultivated plants. Regarding the antioxidant activity, the current study employed ORAC and HORAC assays, whereas most available literature reports are based on DPPH, FRAP and ABTS assays, making direct comparisons challenging. Reported values include DPPH: 158.76 ± 0.82 IC_50_ (μg/mL) and FRAP: 1399 ± 5.01 (mmol Trolox/mg d.w.) [41]; DPPH: 42.00 ± 0.10 μg TE/mL, ABTS: 17.00 ± 0.10 EC_50_ (μg/mL), FRAP: 36.94 ± 0.18 μmol TE/100 mL [10]; DPPH: 26.54 ± 3.8%, FRAP: 28.78 ± 5.2 mg-Trolox/g [43].

Looking deeper into the chemical composition of the water extracts of *S. aethiopis,* attempting to more precisely define its antitumor components, rosmarinic acid appears to be the major dominant compound. Other prevailing compounds are salvianic acid C, gluconic acid, malic acid, citric acid and isopropylmalic acid, as well as salvianic acid A ((R)-3-(3, 4-Dihydroxyphenyl)-2-hydroxypropanoic acid, danshensu) and a compound identified as salvianolic acid K. Salvianolic acids and salvianic acid A are primary active constituents of the traditional Chinese medicinal herb, *Salvia miltiorrhiza* [44,45]—in Chinese, dānshēn, where the alternative name of salvianic acid A, danshensu, comes from.

Consistent with the findings presented in the current work, other studies have also identified rosmarinic acid, salvianolic acids and malic acid in *S. aethiopis* extracts [9,10,46].

Rosmarinic acid (α-o-caffeoyl-3,4-dihydroxy phenyl lactic acid), originally isolated from the herb rosemary (*R. officinalis* L., also known as *S. rosmarinus*) in 1958, and nowadays reported to be present in 39 plant taxa, including *S. aethiopis* [9,10,46], has been extensively studied and validated for its anticancer properties, demonstrating the ability to selectively induce cancer cell apoptosis, suppress tumor growth and inhibit cancer cell proliferation and migration [9,47,48,49]. Breast cancer, ovarian cancer, colorectal cancer and skin cancer, including melanoma and leukemia, are just a few of the cancer types affected [47,49]. Rosmarinic acid has been shown to hold promise for the treatment of lung and hepatocellular cancer as well. Thus, phenolic acid has been demonstrated to have a 50% inhibitory effect on A549 cell proliferation, targeting cyclooxygenase-2, a well-known mediator in tumorigenesis [50]. Furthermore, it was shown that rosmarinic acid decreased the viability of human Hep G2 liver cancer cells in a dose-dependent manner while inducing cancer cell apoptosis, as demonstrated by AO/EB and DAPI staining of the cells and observed changes in the expression of the apoptosis-related proteins Bax and Bcl-2, complemented by the activation of caspase-3 and caspase-9, which prevented the migration and invasion of the Hep G2 cancer cells [51]. Another study further confirmed that rosmarinic acid decreased cell proliferation, inhibited migration and invasion and induced the apoptosis of Hep G2 cells [52]. The above findings are in line with the results presented in the current work, showing extracts of *S. aethiopis* with a high content of rosmarinic acid having detrimental effects on A549 and Hep G2 cancer cells, mediated in Hep G2 cells by cell apoptosis shown by AO/EB and DAPI staining experiments and Annexin-V/propidium iodide-based flow cytometry, accompanied by the decreased migration capacity of the cancer cells as demonstrated by the wound-healing (scratch) assay. Moreover, recent studies have revealed that, beyond its direct anticancer effects, rosmarinic acid is capable of reversing cancer resistance to first-line chemotherapeutics and has a protective effect against the toxicity caused by radiotherapy and chemotherapy, mostly due to its free radical-scavenging ability [53].

There are more than 10 different types of salvianolic acids, known as salvianolic acid A, salvianolic acid B, salvianolic acid C, etc., the most abundant of which are salvianolic acid A and salvianolic acid B. Danshensu and caffeic acid in different combinations are the main building blocks of the various salvianolic acids. Thus, salvianolic acid A is formed by one molecule of danshensu and two molecules of caffeic acid, while salvianolic acid B is formed by three molecules of danshensu and one molecule caffeic acid [54]. Similar to salvianolic acid A, salvianolic acid K also contains one moiety of danshensu and a dimer of caffeic acid, but the difference with salvianolic acid A lies in the specific configuration and the way the moieties are bonded within the salvianolic acid K molecule. [55]. While data on the anticancer activity of salvianolic acid K are missing, Silva et al. [56] report on *Thymus zygis* subsp. zygis, an endemic Portuguese plant, with the major phenolic compounds identified being rosmarinic acid and high amounts of salvianolic acid K and I, whose extracts exhibit significant scavenging activity for ABTS+, hydroxyl (•OH) and nitric oxide (NO) radicals and significant antiproliferative effects against the human colon adenocarcinoma cell line Caco-2, and the human hepatocellular carcinoma cell line Hep G2 used in the present research as well [56]. Data in the scientific literature on the strong antioxidant activity and anticancer effects of the similar-in-structure salvianolic acid A, as well as of salvianolic acid B, are abundant. Regarding the antioxidant activity of salvianolic acid A, it has been shown to not only sequester free radicals directly but to exert cytoprotective effects against oxidative stress by the activation of the Nrf2/HO-1 axis mediated by Akt/mTORC1 activation [57]. Regarding the anticancer activity of salvianolic acid A and salvianolic acid B, it has been shown against numerous types of cancer (amongst which are breast, ovarian and colorectal cancer, squamous cell carcinoma, melanoma and not least liver and lung cancer) and exerted by inducing apoptosis of cancer cells, influencing their cell cycle and inhibiting the proliferative, metastatic and invasive ability of cancer cells by targeting multiple signaling pathways [37,46], only a few of which being the enhancing of caspase-3 activity, downregulating Bcl-2 expression, upregulating Bax expression and disrupting the mitochondrial membrane potential in breast cancer cells [58], inhibiting the AKT/mTOR signaling in hepatocellular carcinoma cells salvianolic acid B [59]) and influencing the PTEN/PI3K/AKT signaling pathway in human lung cancer A549 cells [44]. Recently Tang et al. [60] have shown the anticancer activity of salvianolic acid A on a most aggressive, triple-negative subtype of breast cancer by influencing the polarization of tumor-associated macrophages identified as significant contributors to the growth and metastasis of the cancer. The cytotoxic effects of salvianolic acids on hepatocellular carcinoma and A549 lung cancer cells [44,59] align closely with the current research. Further advantages of salvianolic acids A and B, enhancing their anticancer capacity, are their potency in sensitizing cancer cells to chemotherapeutic agents [61,62] and their ability to inhibit epithelial–mesenchymal transition [44], a process when epithelial cells switch to the mesenchymal cell phenotype and gain migration and invasion capabilities, facilitating tumor progression [63].

Danshensu has also shown anticancer effects, including the suppression of epithelial–mesenchymal transition, the inhibition of tumor angiogenesis and invasion, and the ability to overcome or reduce chemoresistance, while enhancing radiosensitivity in cancer therapy [57,64,65]. Moreover, danshensu exhibits direct antiproliferative activity against A549 lung cancer cells [50] and exerts hepatoprotective effects by inhibiting Hep G2/2.2.15 cell growth, alleviating chronic alcoholic liver disease, counteracting hepatic fibrosis and reducing colon cancer-related liver metastasis [64,66,67,68].

Thus, we believe that the strong cytotoxic effect of the *S. aethiopis* extract against A549 and Hep G2 cancer cell lines was mostly due to rosmarinic acid, being the predominant compound in the extract, and to salvianolic acids and danshensu; all three constituents having demonstrated convincing cytotoxic effects against lung (A549) and hepatocellular cancer (Hep G2) cells, as well as protective effects on the liver tissue (danshensu). Of course, the combination with malic acid, citric acid and gluconic acid, with reported anticarcinogenic properties and activities [69,70,71], likely contributes to the cytotoxic efficiency of the *S. aethiopis* extract.

The results of the present study, limited to in vitro experiments, demonstrating selective anticancer effects on a panel of cell lines through pathological changes in the cancer cells and the inhibition of cancer cell migration, provide a strong foundation for future in vivo experiments using laboratory animals. These in vivo experiments would additionally allow to determine the optimal extract dose, to assess the extract’s bioavailability and pharmacokinetics, to further evaluate the extract’s safety including acute and chronic toxicity, to follow the long-term effects of the extract on tumor progression and recurrence and to explore the combination of the *S. aethiopis* extract with other natural or synthetic compounds to possibly enhance its anticancer activity, all in complex living systems, providing a more comprehensive understanding of the therapeutic potential of *S. aethiopis* and paving the way for its development as an effective anticancer agent.

## 4. Materials and Methods

### 4.1. Plant Materials

**Figure 7:**
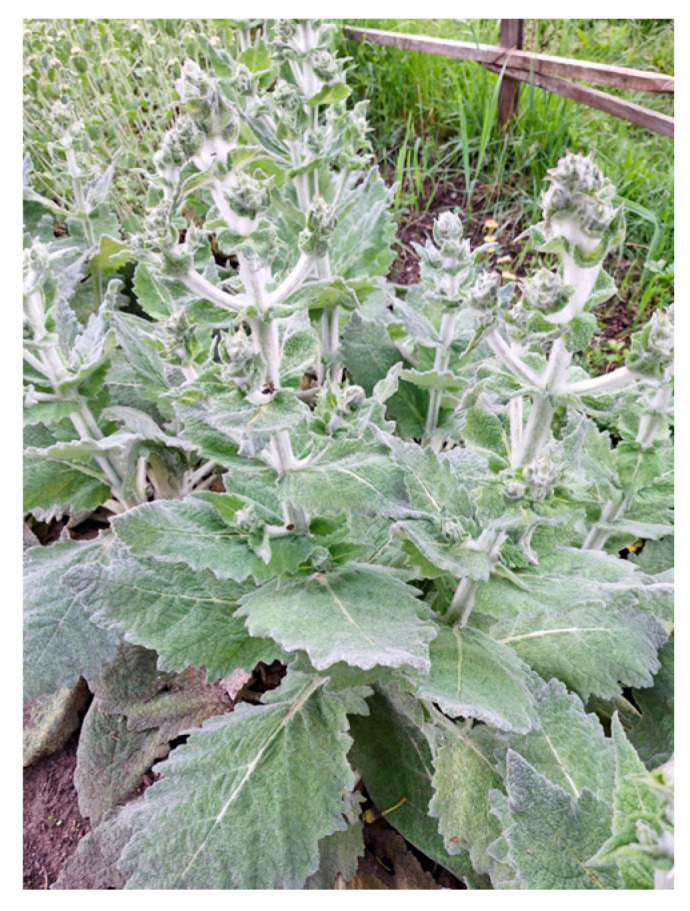
Cultivated *S. aethiopis* in the experimental field.

*Salvia aethiopis* plants were obtained by an in vitro micropropagation technique and cultivated in an open field in Elin Pelin, Sofia region, at an altitude of 618 m above sea level (Figure 7). The aerial parts of the plants (flowers, leaves and stems) were harvested at the end of the vegetation period (June–July, 2023). The collected plant material was dried at room temperature and subsequently used for extract preparation and phytochemical and antitumor analysis.

### 4.2. Phytochemical Analysis

#### 4.2.1. Extraction and Freeze-Dried Procedure

Plant material was first dried and ground to a fine powder using a laboratory mill. For each extraction, 5 g of the powder was steeped in 200 mL of hot water (90 °C) for 15 min. The mixture was then centrifuged at 6000× *g* with an MPW-260R centrifuge (MPW Med. Instruments, Warszawa, Poland), and the supernatants were carefully collected and freeze-dried for 96 h in an Alpha 1–4 LD plus laboratory freeze-drier (manufactured by Martin Christ Gefriertrocknungsanlagen GmbH, Osterode am Harz, Germany). The resulting freeze-dried extracts were subsequently used to evaluate potential antitumor activity.

#### 4.2.2. Quantification of Total Polyphenols and Flavonoid Content

For total polyphenol quantification, the method developed by Singleton and Rossi [72] was employed. Gallic acid was used to establish a standard calibration and the results presented as mg gallic acid equivalents (GAEs) per g dry weight (DW) ± SD.

The total flavonoid content was measured using aluminum chloride (AlCl_3_) as a reagent, following the protocol by Chang et al. [73]. Quercetin dehydrate served as the calibration standard, and the results were expressed as mg quercetin equivalents (QEs) per g DW ± SD.

#### 4.2.3. Evaluation of Antioxidant Activity

The antioxidant potential was assessed by the measurement of oxygen radical absorbance capacity (ORAC) using a microplate reader (FLUOstar OPTIMA; BMG Labtech, Ortenberg, Germany), following the method described by Ou et al. [74], with modifications by Denev et al. [75]. The results are presented as micromole Trolox equivalents (μmol TEs) per gram DW ± SD.

The hydroxyl radical averting capacity (HORAC) of the freeze-dried extract was evaluated based on the protocol of Ou et al. [76]. The results are expressed as micromole gallic acid equivalents (μmol GAEs) per gram DW ± SD.

#### 4.2.4. Liquid Chromatography–High Resolution Mass Spectrometry Analysis (LC-HRMS)

##### Sample Preparation

Each sample (10.0 mg accurately weighted) was mixed with 1.0 mL of 1% formic acid. The solution was extracted with 200 µL of dichloroethane, vortexing it for 5 min, followed by centrifugation at 13,000 rpm for 5 min. Then, 10 µL of the upper phase was used for analysis.

##### LC-HRMS Analysis

Analyses were performed using an Orbitrap IQ-X^®^ hybrid mass spectrometer (ThermoScientific Co., Waltham, MA, USA) coupled with a heated electrospray ionization (HESI) module and Vanquish^®^ Ultra High-Performance Liquid Chromatography (UHPLC) system (ThermoScientific Co., Waltham, MA, USA).

Chromatographic conditions. Separations was carried out on a Nucleodur C18 Isis analytical column (100 × 2.1 mm, 3 µm; Macherey-Nagel, Düren, Germany) under gradient elution at a flow rate of 300 µL/min. The mobile phases were as follows: A, 0.1% formic acid in water and B 0.1% formic acid in acetonitrile and with the gradient program 0% B for 2 min, 0–40% B for 19 min, 40–95% B for 2 min, 95% B for 2 min, 95–0% B for 2 min and 0% B for 3 min.

Mass spectrometry conditions. Full-scan mass spectra were recorded in negative ion mode across the *m*/*z* range of 120–1800 at a resolution of 120,000. Data-dependent reaction monitoring (ddMS2) was performed at a resolution of 17,500 with a 2 amu precursor isolation window. The negative ionization parameters were as follows: spray voltage—3.5 kV; capillary temperature,320 °C; probe heater temperature, 300 °C; sheath gas flow, 20 units; auxiliary gas, 12 units; sweep gas, 3 units (arbitrary values set by IQ-X Tune Software version 3.3 SP3); and S-Lens RF level, 50.00. Nitrogen served as the nebulization and collision gas in the HCD cell. The normalized collision energy was set between 20–35%. Metabolites were quantified using 5 ppm mass tolerance filters against the theoretical *m*/*z* values. Data were acquired and processed using XCalibur^®^ software (Software version 4.1, ThermoScientific Co., Waltham, MA, USA).

### 4.3. Antitumor Activity

#### 4.3.1. Cell Lines

The following permanent cell lines were obtained from the American Type Culture Collection (ATCC, Rockville, MD, USA): murine embryonic fibroblasts (BALB/3T3, CCL-163), human lung carcinoma (A549, CRM-CCL-185), human prostate adenocarcinoma (PC-3, CRL-1435) and human hepatocellular carcinoma (Hep G2, HB-8065). The human keratinocyte cell line HaCaT (CVCL_0038) was acquired from the CLS Cell Lines Service (Cytion, Eppelheim, Germany) and employed as a non-tumorigenic control to assess the selectivity of the anticancer effects. All cell lines were cultured in Dulbecco Modified Eagle Medium (DMEM), supplemented with 10% fetal bovine serum, 100 U/mL penicillin and 100 μg/mL streptomycin. Cultures were kept at 37 °C in a humidified atmosphere containing 5% CO_2_ (HEPA Class 100, Thermo Scientific, Waltham, MA, USA). Upon reaching confluence, cell monolayers were detached using a 0.05% trypsin–0.02% ethylendiaminotetraacetic acid (EDTA) solution, and cells in the exponential growth phase were used for subsequent experiments.

#### 4.3.2. BALB/3T3 Neutral Red Uptake Assay

The cytotoxicity of the *S. aethiopis* extract was evaluated by the BALB/3T3 Neutral Red Uptake assay. BALB/3T3 cells were seeded in 96-well flat-bottomed plates at a density of approximately 1 × 10^5^ cells/mL in DMEM supplemented with 10% FBS (100 µL/well) and incubated overnight at 37 °C with 5% CO_2_. The cells were then exposed to different concentrations of the extract (ranging from 15.6 to 2000 μg/mL) and incubated for 24 h, 48 h and 72 h. Following treatment, the wells were washed twice with PBS, and 100 µL of DMEM containing neutral red dye (50 μg/mL) was added to each well. After 2 h of incubation under standard culture conditions, the neutral red medium was removed, the wells were washed with PBS, and a desorbing solution of ethanol/water/acetic acid (50:49:1) was added. The plates were incubated for 10 min, and absorbance was measured by a TECAN microplate reader (Sunrise^TM^, Grödig/Salzburg, Austria) at 540 nm.

#### 4.3.3. Assessment of Antiproliferative Activity

The antiproliferative activity of the *S. aethiopis* extract on cancer cell lines was evaluated using the standard MTT colorimetric assay. Non-tumorigenic HaCaT cells were used as a reference to determine the selectivity of the extract toward cancer cells. Cells were detached with 0.25% Trypsin–EDTA, counted in a hemocytometer and diluted to a concentration of 1 × 10^4^ cells/mL before being plated in 96-well plates. The cells were incubated overnight at 37 °C in a humidified atmosphere containing 5% CO_2_. Following overnight incubation at 37 °C in a humidified 5% CO_2_ atmosphere, the cells were treated with *S. aethiopis* extract at concentrations ranging from 15.6 to 1000 μg/mL, with untreated cells as controls. Each concentration was tested in quintuplicate. After 72 h exposure, the cells were rinsed with PBS, followed by the addition of 100 μL of MTT solution (5 mg/mL in DMEM) to each well. After a further 3 h incubation at 37 °C, the supernatants were removed and replaced with 100 μL of DMSO/ethanol solution (1:1 ratio) to solubilize the purple formazan crystals. Absorbance was measured at 570 nm using a TECAN Sunrise^TM^ ELISA plate reader (Grodig/Salzburg, Austria). Cell viability was expressed as a percentage relative to the untreated control.

#### 4.3.4. Cytopathological Studies—Live/Dead Staining with Acridine Orange and Ethidium Bromide

To evaluate cytopathological changes in Hep G2 hepatocellular carcinoma cells treatment with *S. aethiopis* extract, dual fluorescent live/dead staining with acridine orange (AO) and ethidium bromide (EtBr) was performed. Cells were plated on sterile glass coverslips in 24-well plates at a density of 1 × 10^5^ cells per well and allowed to adhere overnight. The next day, cells were exposed to the extract at its IC_50_ concentration for 72 h. The control groups included untreated HepG2 cells (negative control) and doxorubicin-treated cells (a positive control) cultured under the same conditions. Post-treatment, the coverslips were removed, gently rinsed with phosphate-buffered saline (PBS) and stained with a mixture of AO (10 μg/mL in PBS) and EtBr (10 μg/mL each in PBS). The stained cells were then mounted on glass slides and immediately visualized under a Leica DM fluorescence microscope (5000B, Wetzlar, Germany) before fluorescence fading could occur.

#### 4.3.5. Nuclear Morphology Analysis by DAPI Staining

The nuclear morphology of Hep G2 cells treated with *S. aethiopis* extract was investigated by staining them with the DNA-binding fluorescent dye 4′,6-diamidino-2-phenylindole (DAPI). Hep G2 cells were cultivated and treated as described in the previous section. Following incubation, the glass coverslips were washed twice with phosphate-buffered saline (PBS) to remove detached cells. The cells were then fixed with ice-cold methanol, and DAPI staining was performed according to the manufacturer’s protocol. Samples of treated and untreated (control) cells were mounted with Mowiol on microscope slides and stored in the dark until examination with a Leica fluorescence microscope (DM 5000B, Wetzlar, Germany).

#### 4.3.6. Flow Cytometric Analysis

The cell cycle progression and apoptosis of Hep G2 hepatocellular carcinoma cells following treatment with *S. aethiopis* extract were evaluated by flow cytometry. Cells were seeded in 6-well plates, incubated for 24 h and then treated with *S. aethiopis* extract at its IC_50_ concentration for 72 h. Untreated Hep G2 cells served as controls. Following the treatment period, cells were harvested using Trypsin–EDTA solution (Sigma-Aldrich, Burlington, MA, USA) and washed twice with cold PBS by centrifugation (1000 rpm, 10 min), and the resulting cell pellets were collected for the s cell cycle and apoptosis analyses, as detailed in subsequent sections.

#### 4.3.7. Cell Cycle Assay

Hep G2 cells, untreated controls and those treated with *S. aethiopis* extract, were washed with PBS and fixed with 70% ice-cold ethanol, added dropwise under continuous vortexing. The cells were then stored overnight at −20 °C. The next day, the fixed cells were washed with PBS and incubated with RNase A (20 µg/mL; Roche Diagnostics GmbH, Mannheim, Germany) for 30 min. Subsequently, the cells were stained with PI (20 µg/mL) and analyzed by flow cytometry (Becton Dickinson, BD Biosciences, San Jose, CA, USA) to assess the distribution of cell cycle phases. The proportions of cells in G1, S and G2-M were determined with FlowJo™ v10.8 software (BD Biosciences, San Jose, CA, USA). Data are expressed as the mean ± standard error of the mean from three independent replicates.

#### 4.3.8. Apoptosis Detection Assay

Apoptosis was assessed using the Annexin V-FITC Apoptosis Detection Kit (sc-4252 AK, Santa Cruz Biotechnology, Inc., Dallas, TX, USA) following the manufacturer’s protocol. Control and treated cells were stained and incubated for 15 min at room temperature, then analyzed by a flow cytometer (BD FACSCalibur™). The proportions of viable, early apoptotic, late apoptotic and necrotic cells were quantified with FlowJo software (BD Biosciences, San Jose, CA, USA).

### 4.4. Statistical Analysis

Data are expressed as the mean ± standard error of the mean (SEM). Statistical analyses were conducted using GraphPad Prism 9.0 software. A one-way analysis (ANOVA) was applied, with the Shapiro–Wilks test to assess data normality, followed by Tukey’s post hoc test for multiple comparisons. A *p*-value of less than 0.05 (*p* < 0.05) was considered statistically significant.

## 5. Conclusions

This study demonstrates that aqueous extracts from cultivated *Salvia aethiopis* possess potent anticancer properties against hepatocellular, liver and prostate carcinoma, with the most pronounced effects against liver cancer. The extract selectively inhibits hepatocellular carcinoma cell proliferation by induced apoptosis, necrosis and altered cell cycle progression, while maintaining a low toxicity to non-cancerous cells. The anticancer activity is underlined by a high content of phenolics in the *S. aethiopis* extracts, including rosmarinic acid and salvianolic acids, as well as salvianolic acid K and danshensu, known for their strong anticancer activity. These findings underscore the potential of *S. aethiopis* as a valuable source of bioactive compounds for therapeutic applications, warranting further investigation into its mechanism of action and in vivo efficacy.

## Figures and Tables

**Table 1:** Total polyphenol and flavonoid content and antioxidant activity of freeze-dried extract from in vitro cultivated *Salvia aethiopis*.

Sample	Total Polyphenols (mg GAE/g DW)	Total Flavonoids (mg QE/g DW)	ORAC (µmol TE/g DW)	HORAC (µmol GAE/g DW)
*S. aethiopis* (cultivated)	110.03 ± 0.7	7.88 ± 0.25	3677.9 ± 24.8	889.6 ± 14.3

**Table 2:** Identification of phytochemical compounds of *Salvia aethiopis* extract by LC-HRMS in negative mode. The numbers in parentheses indicate the corresponding compound identified either in a scientific publication or by database matching.

Peak	RT (min)	Experimental *m*/*z* Error (ppm)	Molecular Formula	MS^2^ Fragments *m*/*z*, (R.I., %)	Proposed Compound
**1**	0.48	195.0483 (−13.7)	C_6_H_12_O_7_	71.0125 (8), 75.0073 (51), 85.0280 (7), 87.0071 (19), 99.0070 (14), 105.0176 (21), 129.0173 (32), 147.0275 (7), 159.0273 (6), 177.0378 (11)	Gluconic acid [25,26]
**2**	0.54	341.1038 (−14.9)	C_12_H_22_O_11_	59.0127 (76), 71.0125 (62), 89.0229 (100), 95.0122 (9), 101.0227 (70),113.0226 (59), 119.0331 (39), 131.0329 (9), 143.0328 (17), 161.0430 (15), 179.0534 (44)	Disaccharide [25,27]
**3**	0.56	133.0122 (−14.9)	C_4_H_6_O_5_	71.0124 (38), 72.9916 (15), 89.0227 (7), 115.0017 (100)	Malic acid [10,25]
**4**	1.20	191.0172 (−13.8)	C_6_H_8_O_7_	85.0279 (29), 99.0070 (6), 111.0069 (100), 117.0173 (21), 129.0172 (14), 154.9962 (16), 173.0065 (27)	Citric acid [25]
**5**	4.55	197.0429 (−13.5)	C_9_H_10_O_5_	72.9917 (55), 123.0433 (78), 134.0353 (13), 135.0432 (100), 179.0326 (58)	Danshensu (3-(3,4-Dihydroxyphenyl)-2-hydroxypropanoic acid) [28]
**6**	5.94	315.0679 (−13.5)	C_13_H_16_O_9_	153.0170 (34), 152.0092 (98), 109.0278 (36), 108.0200 (90)	Protocatechuic acid hexoside [25,29]
**7**	6.91	175.0586 (−14.9)	C_7_H_12_O_5_	85.0643 (32), 113.0589 (36), 115.0381 (87), 131.0693 (8), 157.0482 (8), 175.0585 (90)	Isopropylmalic acid [25,30]
**8**	8.29	475.1754 (−14.0)	C_21_H_32_O_12_	71.0122 (12), 85.0277 (12), 101.0225 (9), 103.0380 (8) 113.0223 (72), 149.0583 (6), 161.0427 (8), 329.1194 (8)	2-(3-Hydroxy-4-methoxyphenyl)ethyl-*O*-(rhamnosyl)glucopyranoside
**9**	9.43	377.0832 (−13.4)	C_18_H_18_O_9_	133.0274 (5), 135.0432 (17), 137.02220 (7), 161.0220 (100), 179.0323 (17), 197.0427 (31), 359.0721 (22)	Salvianic acid C
**10**	9.92	569.1068 (−14.4)	C_24_H_26_O_16_	111.0069 (23), 121.0275 (9), 129.0173 (100.00), 138.0303 (5), 147.0276 (28), 153.0532 (28), 173.0063 (26), 182.0193(5), 191.0169 (16), 197.0424 (96), 265.0676 (23), 327.0671 (33), 371.0561 (52), 389.0666 (21)	Ester of salvianic acid C and 2,3,4,5-tetrahydroxyhexanedioic acid
**11**	12.02	651.1111 (−13.9)	C_28_H_28_O_18_	113.0226 (35), 175.0221 (9), 193.0323 (23), 284.0286 (6), 285.0367 (6), 289.0525 (4), 299.0520 (15), 351.0520 (100)	*O*-(*O*-Glucuronyl-*O*-glucuronide)methoxylated flavonoid
**12**	13.09	359.0722 (−14.5)	C_18_H_16_O_8_	123.0434 (5), 132.0202 (7), 135.0432 (13), 161.0220 (100), 179.0324 (18), 197.0427 (30)	Rosmarinic acid [10]
**13**	13.76	475.0817 (−17.3)	C_22_H_20_O_12_	113.0225 (26), 175.0220 (7), 284.0284 (32), 299.0516 (100)	Methoxylated flavonoid-*O*-glucoronide [25]
**14**	13.96	207.0634 (−14.2)	C_11_H_12_O_4_	135.0429 (100), 136.0510 (5), 162.0299 (6), 163.0375 (11), 164.0456 (8), 174.0295 (9), 177.0529, 192.0399 (15)	Unknown
**15**	14.39	629.2346 (−16.2)	C_29_H_42_O_15_	153.0533 (5), 165.1258 (5), 196.0346 (5), 197.0423 (47), 207.1357 (11), 209.1150 (30), 211.0580 (15), 224.0293 (6), 225.1462 (8), 239.0523 (81), 251.1250 (8), 269.1352 (100), 285.0577 (6), 299.0731 (27), 359.0930 (5)	Unknown
**16**	14.87	813.1404 (−14.0)	C_37_H_34_O_21_	113.0225 (35), 135.0430 (45), 161.0218 (39), 175.0219 (15), 179.0322 (80), 193.0322 (29), 203.0318 (6), 245.0420 (18), 284.0287 (14), 285.0366 (17), 289.0526 (5), 299.0521 (45), 333.0417 (21), 337.0517 (14), 351.0517 (55), 355.0616 (5), 513.0811 (100), 633.0996 (6)	*O*-(*O*-Caffeoyl-*O*-glucuronyl-*O*-glucuronide)methoxylated flavonoid
**17**	15.04	555.1065 (−14.2)	C_27_H_24_O_13_	133.0274 (5), 135.0431 (80), 161.0220 (100), 179.0322 (25), 197.0426 (33), 295.0573 (10), 359.0718 (97), 401.0821 (7), 493.1060 (19)	Salvianolic acid K [27]
**18**	15.65	857.1658 (−13.8)	C_39_H_38_O_22_	113.0225 (26), 149.0220 (16), 164.0452 (31), 175.0218 (10), 179.0685 (5), 223.0579 (79), 284.0287 (16), 285.0362 (6), 299.0521 (41), 333.0417 (25), 351.0516 (7), 399.0880 (5), 557.1068 (100), 633.1000 (6)	*O*-(*O*-Sinapoyl-*O*-glucuronyl-*O*-glucuronide)methoxylated flavonoid
**19**	16.03	827.1561 (−13.2)	C_38_H_36_O_21_	113.0225 (27), 134.0351 (37), 149.0584 (7), 175.0217 (10), 193.0473 (87), 284.0284 (16), 285.0365 (7), 299.0518 (44), 333.0413 (27), 351.0527 (7), 369.0772 (5), 527.0952 (100), 633.0990 (6)	*O*-(*O*-Feruloyl-*O*-glucuronyl-*O*-glucuronide)methoxylated flavonoid
**20**	17.49	327.2132 (−12.0)	C_18_H_32_O_5_	137.0952 (13), 171.1001 (100), 201.1100 (8), 211.1310 (18), 221.1160 (5), 229.1415 (11)	Dihydroxy-oxo-octadecenoic acid [10,25]
**21**	17.58	313.0675 (−11.7)	C_17_H_14_O_6_	133.0273 (14), 151.0376 (7), 161.0219 (100)	2-(3,4-dihydroxyphenyl)ethenyl 3-(3,4-dihydroxyphenyl)prop-2-enoate

**Table 3:** Half-maximal inhibitory concentration (IC_50_) of *S. aethiopis* extract in human cancer cell lines and selectivity index (SI) relative to non-cancerous cells, as determined by the MTT assay. IC_50_ values are expressed as mean ± standard deviation (SD).

Cell Lines	IC_50_ (μg/mL)	Selectivity Index (SI)
HaCaT (non-tumorigenic)	>2000	-
A549 (lung carcinoma)	683.4 ± 7.5	>2.9
Hep G2 (hepatocellular carcinoma)	353.8 ± 21.8	>5.7
PC-3 (prostate carcinoma)	720.1 ± 15.2	>2.8

## Data Availability

All data are contained in the manuscript.

## Supplementary Materials

The following supporting information can be downloaded at: https://www.mdpi.com/article/10.3390/molecules30071427/s1, Figure S1. UPLC-DAD chromatogram at 280 nm of extract of *Salvia aethiopis*; Figure S2. The total ion chromatogram (TIC) of the extract of *Salvia aethiopis*; Figure S3. MS/MS spectrum of compound **1** (gluconic acid) using ESI in negative ionization mode; Figure S4. MS/MS spectrum of compound **2** (disaccharide) using ESI in negative ionization mode; Figure S5. MS/MS spectrum of compound **3** (malic acid) using ESI in negative ionization mode; Figure S6. MS/MS spectrum of compound **4** (citric acid) using ESI in negative ionization mode; Figure S7. MS/MS spectrum of compound **5** (danshensu) using ESI in negative ionization mode; Figure S8. MS/MS spectrum of compound **6** (protocatechuic acid hexoside) using ESI in negative ionization mode; Figure S9. MS/MS spectrum of compound **7** (isopropylmalic acid) using ESI in negative ionization mode; Figure S10. MS/MS spectrum of compound **8** (2-(3-hydroxy-4-methoxyphenyl)ethyl-*O*-(rhamnosyl)glucopyranoside) using ESI in negative ionization mode; Figure S11. MS/MS spectrum of compound **9** (salvianic acid C) using ESI in negative ionization mode; Figure S12. MS/MS spectrum of compound **11** (ester of salvianic acid C and 2,3,4,5-tetrahydroxyhexanedioic acid) using ESI in negative ionization mode; Figure S13. MS/MS spectrum of compound **11** (*O*-(*O*-glucuronyl-*O*-glucuronide)methoxylated flavonoid) using ESI in negative ionization mode; Figure S14. MS/MS spectrum of compound **12** (rosmarinic acid) using ESI in negative ionization mode; Figure S15. MS/MS spectrum of compound **13** (methoxylated flavonoid-*O*-glucuronide) using ESI in negative ionization mode; Figure S16. MS/MS spectrum of compound **14** (unknown) using ESI in negative ionization mode; Figure S17. MS/MS spectrum of compound **15** (unknown) using ESI in negative ionization mode; Figure S18. MS/MS spectrum of compound **16** (*O*-(*O*-caffeoyl-*O*-glucuronyl-*O*-glucuronide)methoxylated flavonoid) using ESI in negative ionization mode; Figure S19. MS/MS spectrum of compound **17** (salvianolic acid K) using ESI in negative ionization mode; Figure S20. MS/MS spectrum of compound **18** (*O*-(*O*-sinapoyl-*O*-glucuronyl-O-glucuronide)methoxylated flavonoid) using ESI in negative ionization mode; Figure S21. MS/MS spectrum of compound **19** (*O*-(*O*-feruloyl-*O*-glucuronyl-*O*-glucuronide)methoxylated flavonoid) using ESI in negative ionization mode; Figure S22. MS/MS spectrum of compound **20** (dihydroxy-oxooctadecenoic acid) using ESI in negative ionization mode; Figure S23. MS/MS spectrum of compound **21** (2-(3,4-dihydroxyphenyl)ethenyl 3-(3,4-dihydroxyphenyl)prop-2-enoate) using ESI in negative ionization mode; Figure S24. Molecular structure of several compounds of the extract of *Salvia aethiopis*.
