# Supplementary material for: Phytochemical Profile, Antioxidant Capacity and Anticancer Potential of Water Extracts from In Vitro Cultivated Salvia aethiopis
Source: Molecules. 2025 Mar 23;30(7):1427. doi: 10.3390/molecules30071427 (PMC11990555; doi:10.3390/molecules30071427)
Supplement: Supplementary file 1 [file molecules-30-01427-s001.zip › molecules-3531794-supplementary.pdf]

Supplementary Material

Phytochemical profile, antioxidant capacity and anticancer potential of water extracts from *in vitro* cultivated *Salvia aethiopis*

Krasimira Tasheva[1], Inna Sulikovska[2], Ani Georgieva[2], Vera Djeliova[3], Vesela Lozanova[4], Anelia Vasileva[4], Ivaylo Ivanov[4], Petko Denev[5], Maria Lazarova[6], Valya Vassileva[1*], Polina Petkova-Kirova[6]

[1]Institute of Plant Physiology and Genetics, Bulgarian Academy of Sciences, Acad. G. Bonchev Str., 21, 1113 Sofia, Bulgaria; E-mails: e-mail: krasitasheva@abv.bg (K.T.); valyavassileva@bio21.bas.bg (V.V.);
[2]Department of Pathology, Institute of Experimental Morphology, Pathology and Anthropology with Museum, Bulgarian Academy of Sciences Sofia, Bulgaria; inna_sulikovska@ukr.net (I.S.); georgieva_any@abv.bg (A.G.);
[3]Department of Molecular biology of cell cycle, Institute of Molecular Biology "Acad. R. Tsanev", Bulgarian Academy of Sciences, Acad. G. Bonchev Str, bl. 21 Sofia 1113, Bulgaria e-mail: vera@bio21.bas.bg (V.D.);
[4]Department of Medical Chemistry and Biochemistry, Medical University – Sofia, Sofia, Bulgaria; E-mails: vlozanova@medfac.mu-sofia.bg (V.L.); avasileva@medfac.mu-sofia.bg (A.V.); iivanov@medfac.mu-sofia.bg (I.I.);
[5]Institute of Organic Chemistry with Centre of Phytochemistry, Bulgarian Academy of Sciences, Laboratory of Biologically Active Substances, Plovdiv, Bulgaria; E-mails: petko.denev@orgchm.bas.bg (P.D.);
[6]Department of Synaptic Signaling and Communication, Institute of Neurobiology, Bulgarian Academy of Sciences, Sofia, Bulgaria; E-mails: m.lazarova@gmail.com (M.L.); kirovaps@yahoo.com (P.P.-K.).
* Correspondence: valyavassileva@bio21.bas.bg (V.V.).

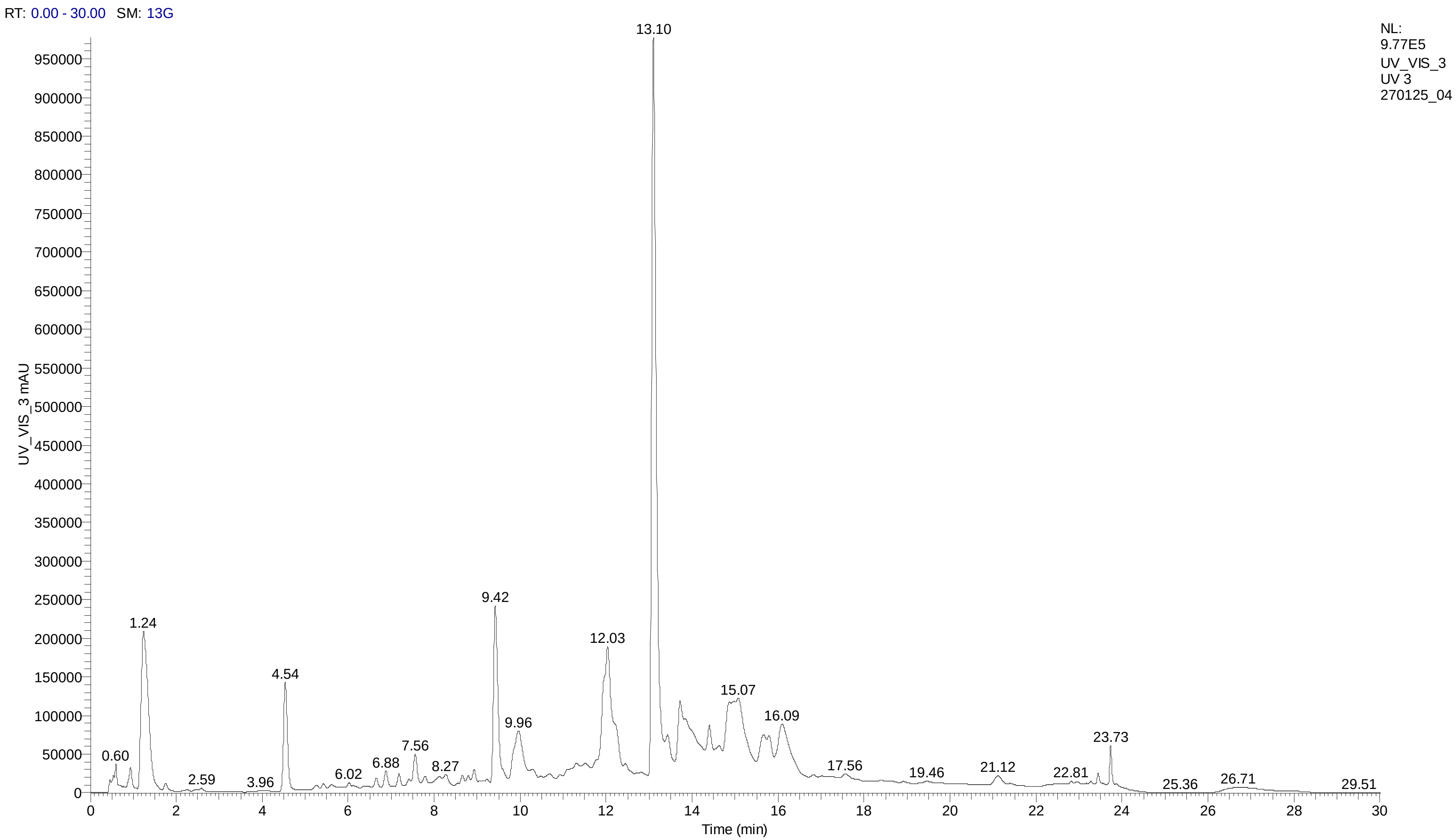

Figure S1. UPLC-DAD chromatogram at 280 nm of extract of *Salvia aethiopis*.

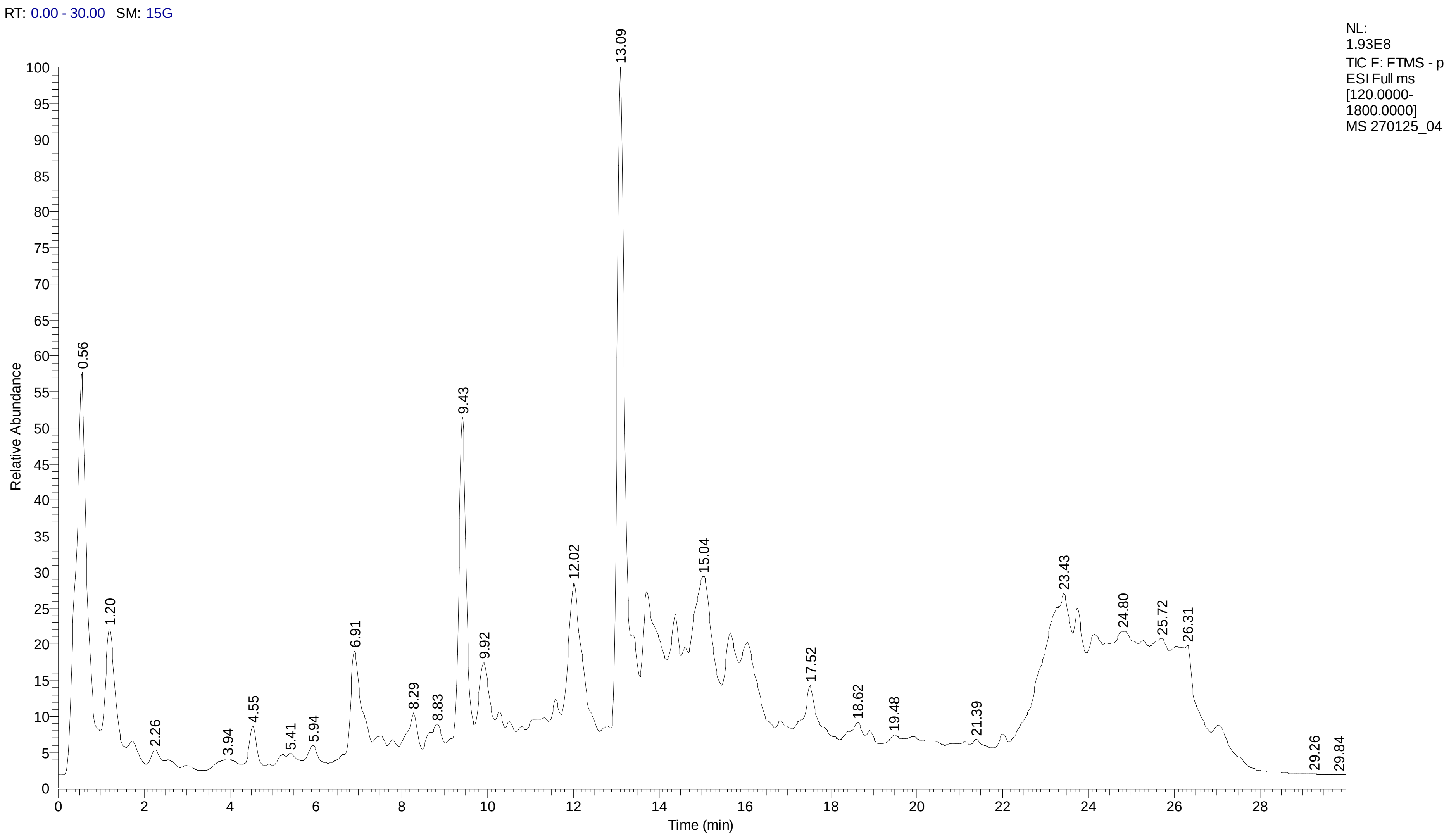

Figure S2. The total ion chromatogram (TIC) of the extract of *Salvia aethiopis*.

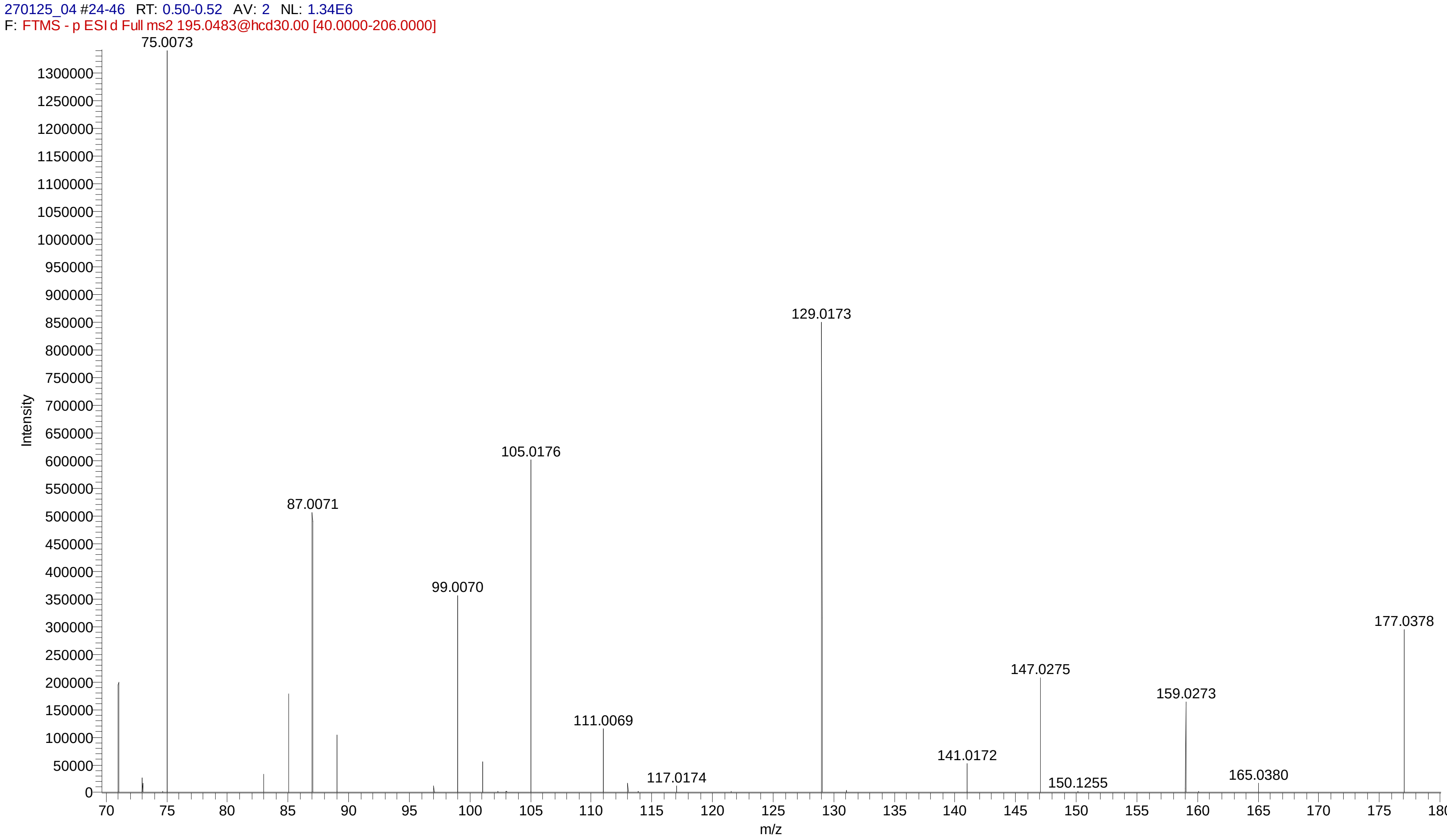

Figure S3. MS/MS spectrum of compound **1** (Gluconic acid) using ESI in negative ionization mode.

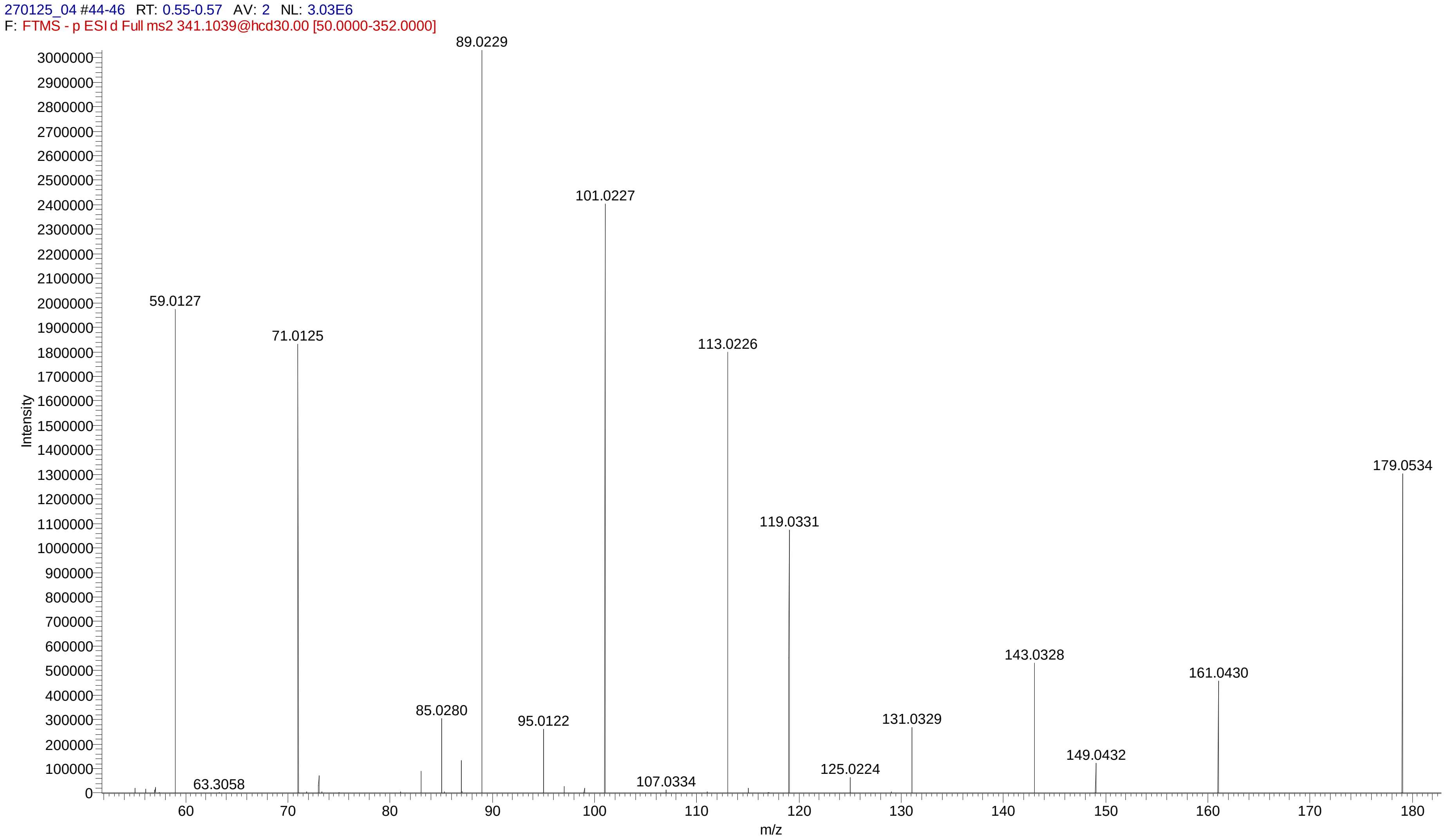

Figure S4. MS/MS spectrum of compound **2** (Disaccharide) using ESI in negative ionization mode.

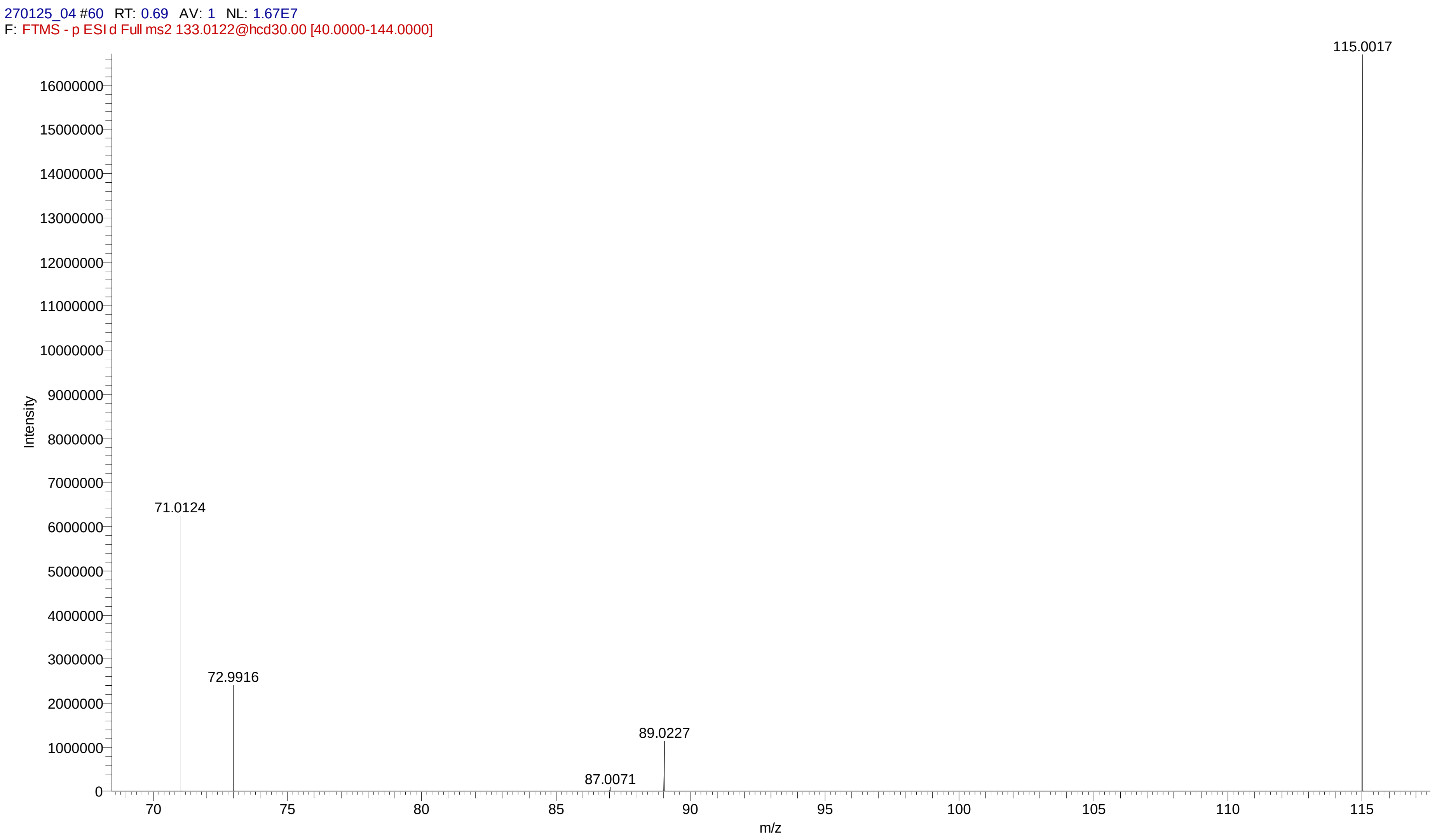

Figure S5. MS/MS spectrum of compound **3** (Malic acid) using ESI in negative ionization mode.

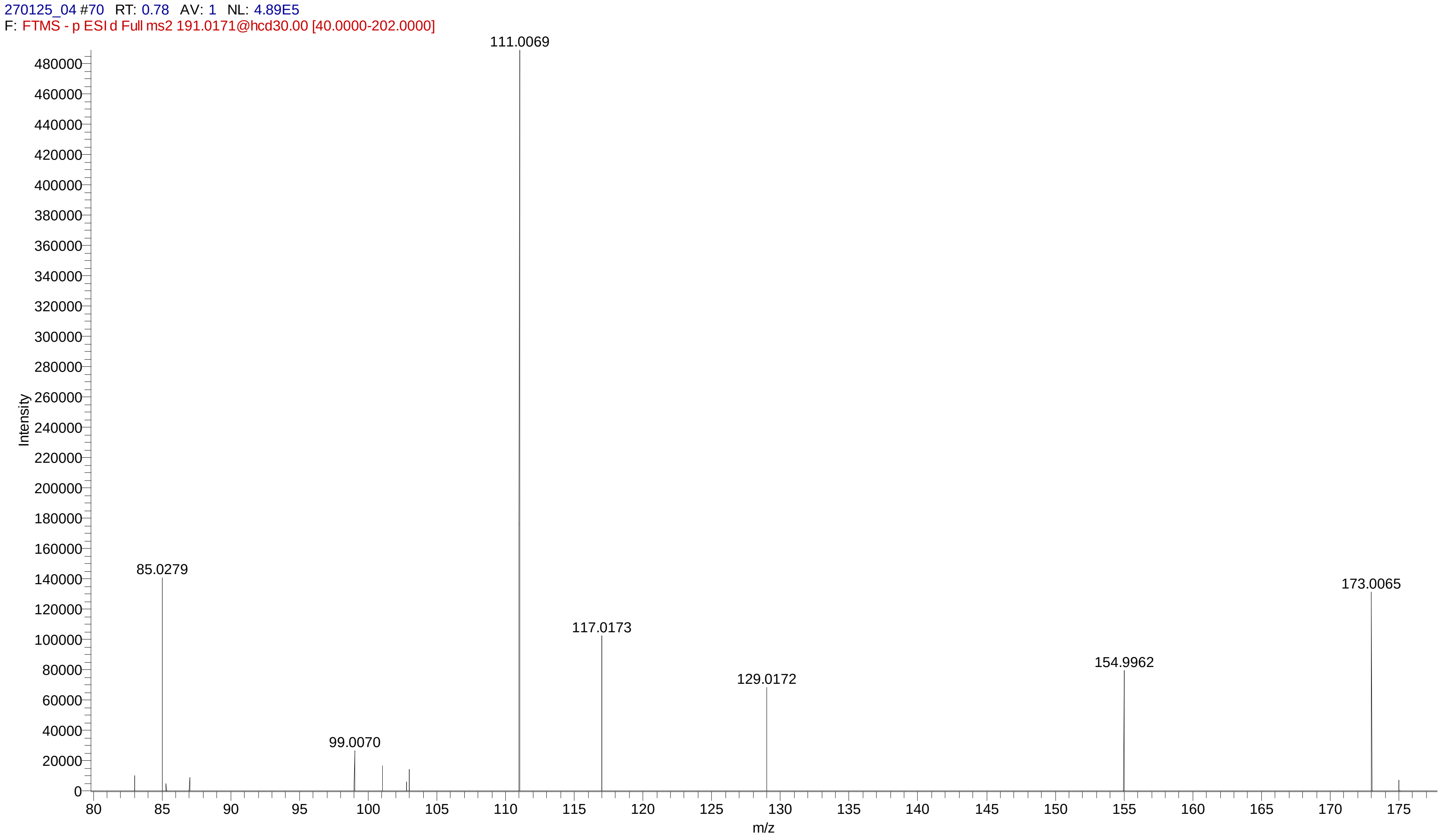

Figure S6. MS/MS spectrum of compound **4** (Citric acid) using ESI in negative ionization mode.

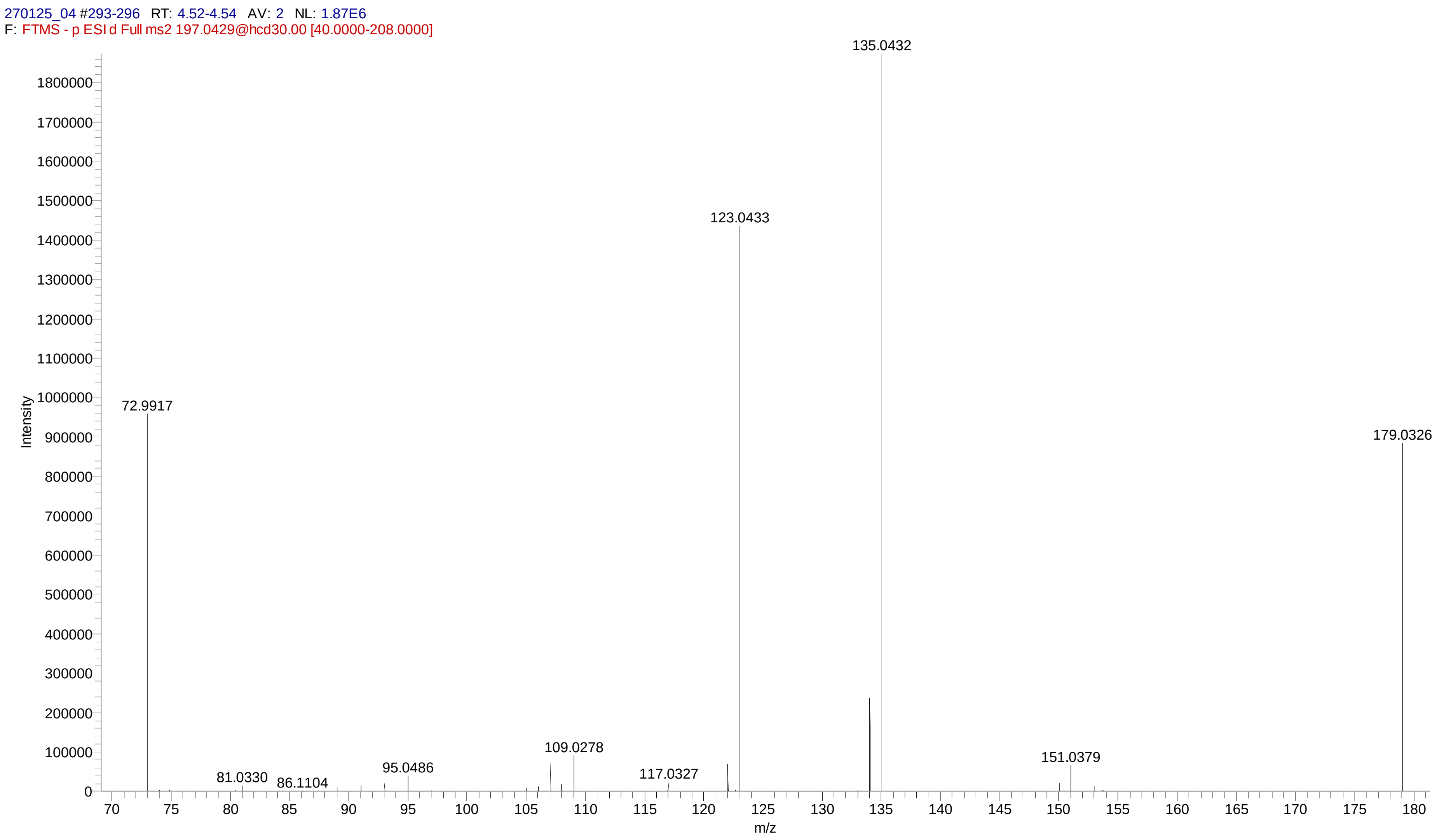

Figure S7. MS/MS spectrum of compound **5** (Danshensu) using ESI in negative ionization mode.

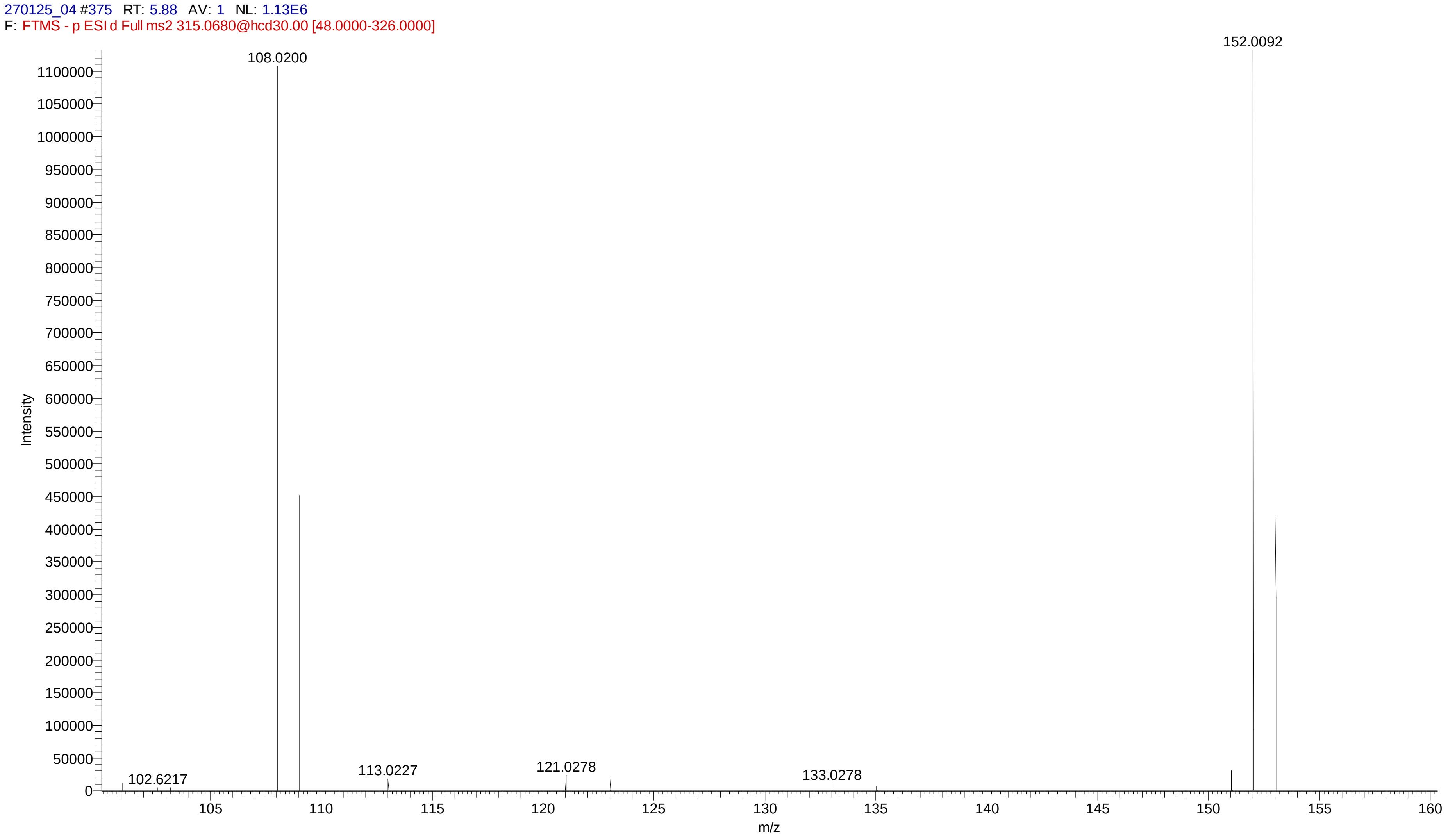

Figure S8. MS/MS spectrum of compound **6** (Protocatechuic acid hexoside) using ESI in negative ionization mode.

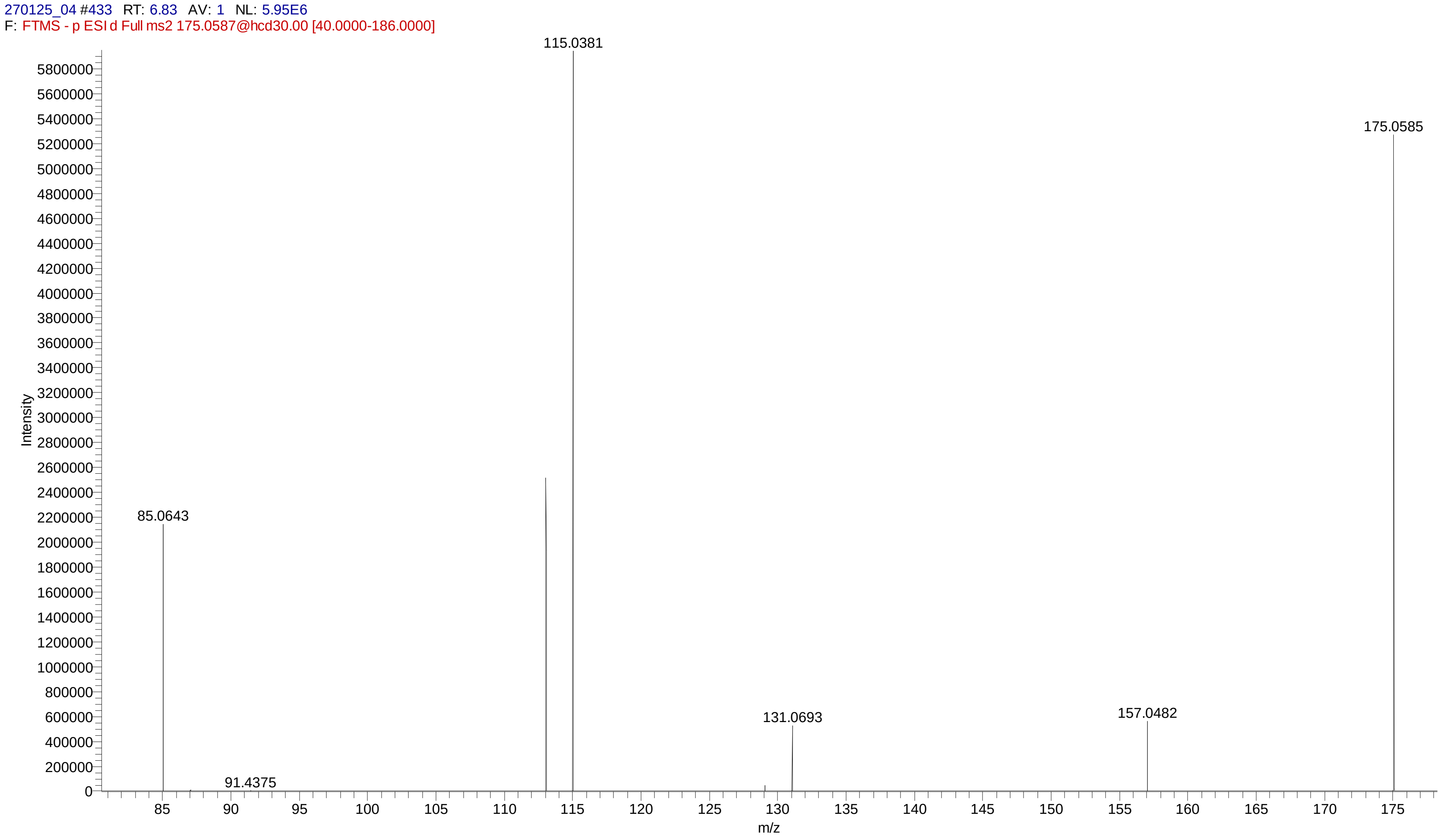

Figure S9. MS/MS spectrum of compound **7** (Isopropylmalic acid) using ESI in negative ionization mode.

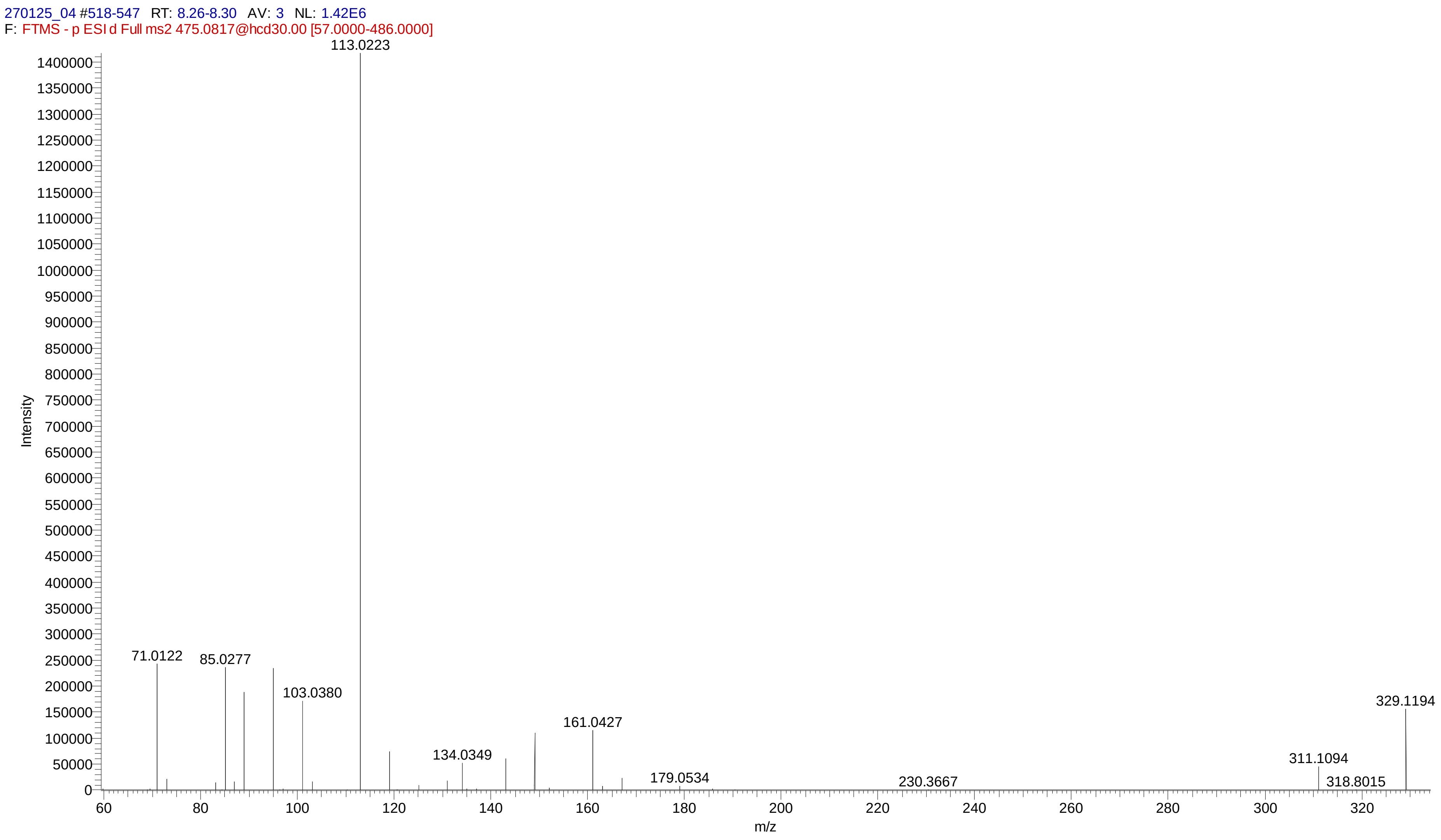

Figure S10. MS/MS spectrum of compound **8** (2-(3-Hydroxy-4-methoxyphenyl)ethyl-O-(rhamnosyl)glucopyranoside) using ESI in negative ionization mode.

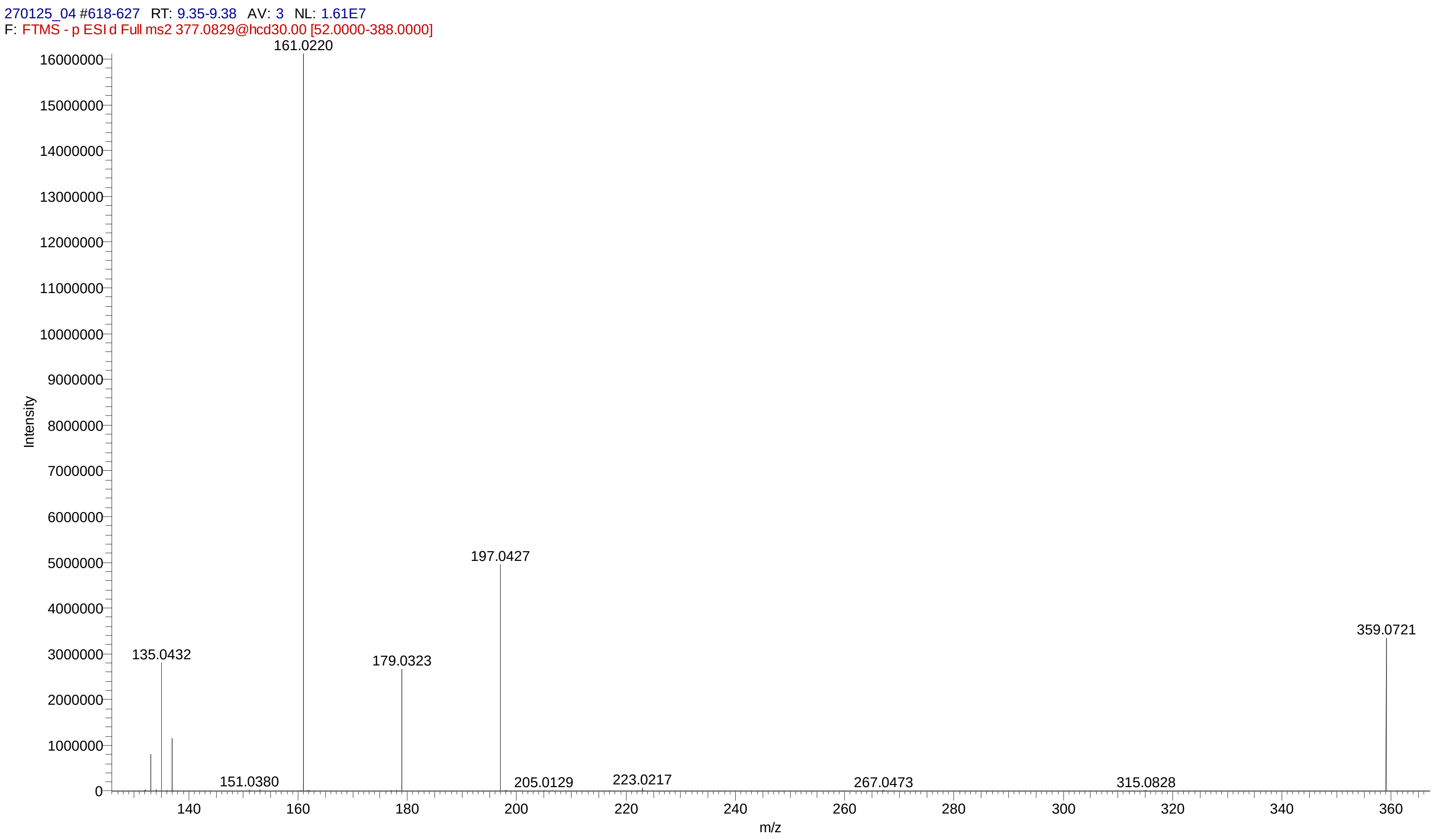

Figure S11. MS/MS spectrum of compound **9** (Salvianic acid C) using ESI in negative ionization mode.

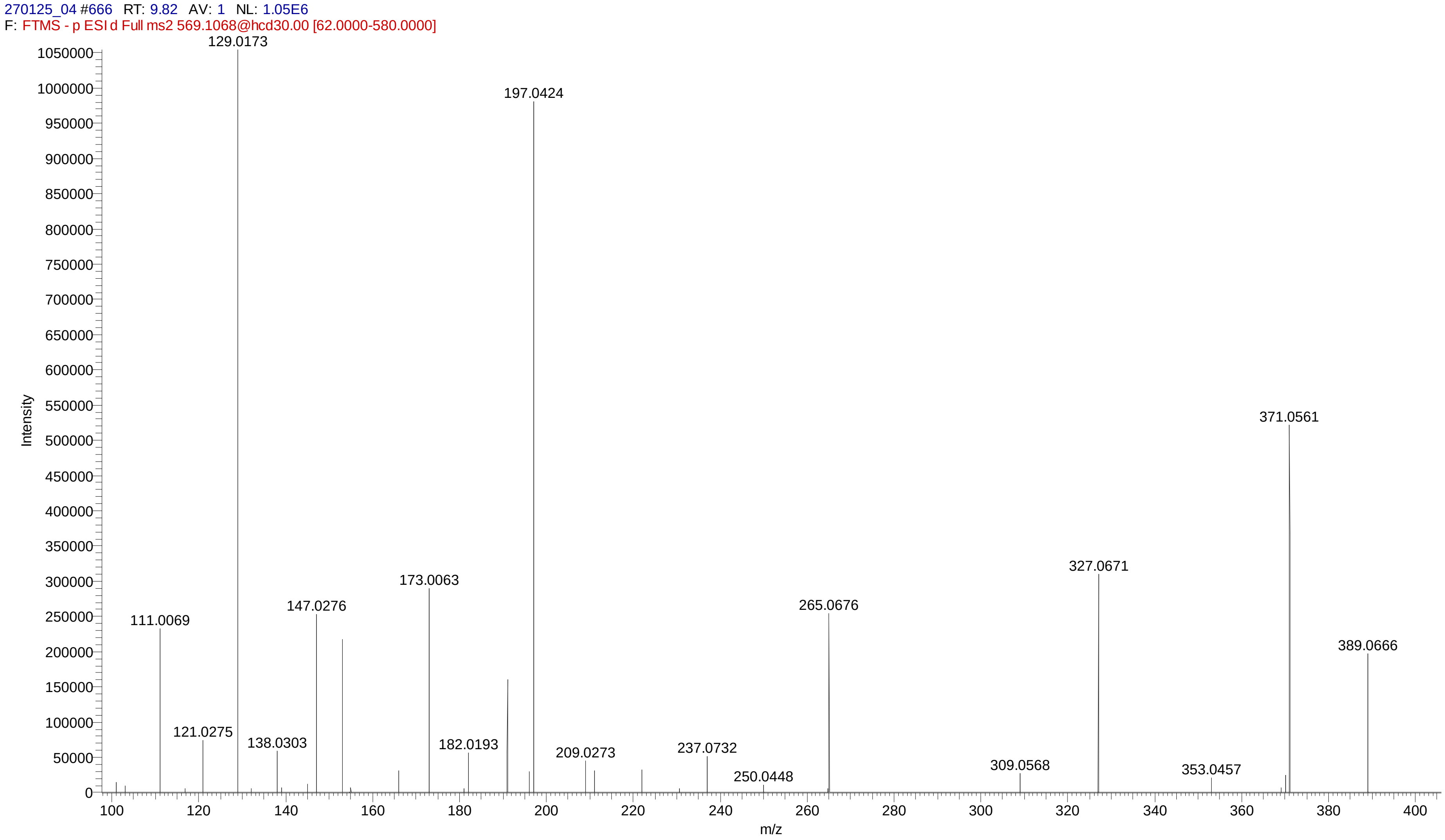

Figure S12. MS/MS spectrum of compound **10** (Ester of salvianic acid C and 2,3,4,5-tetrahydroxyhexanedioic acid) using ESI in negative ionization mode.

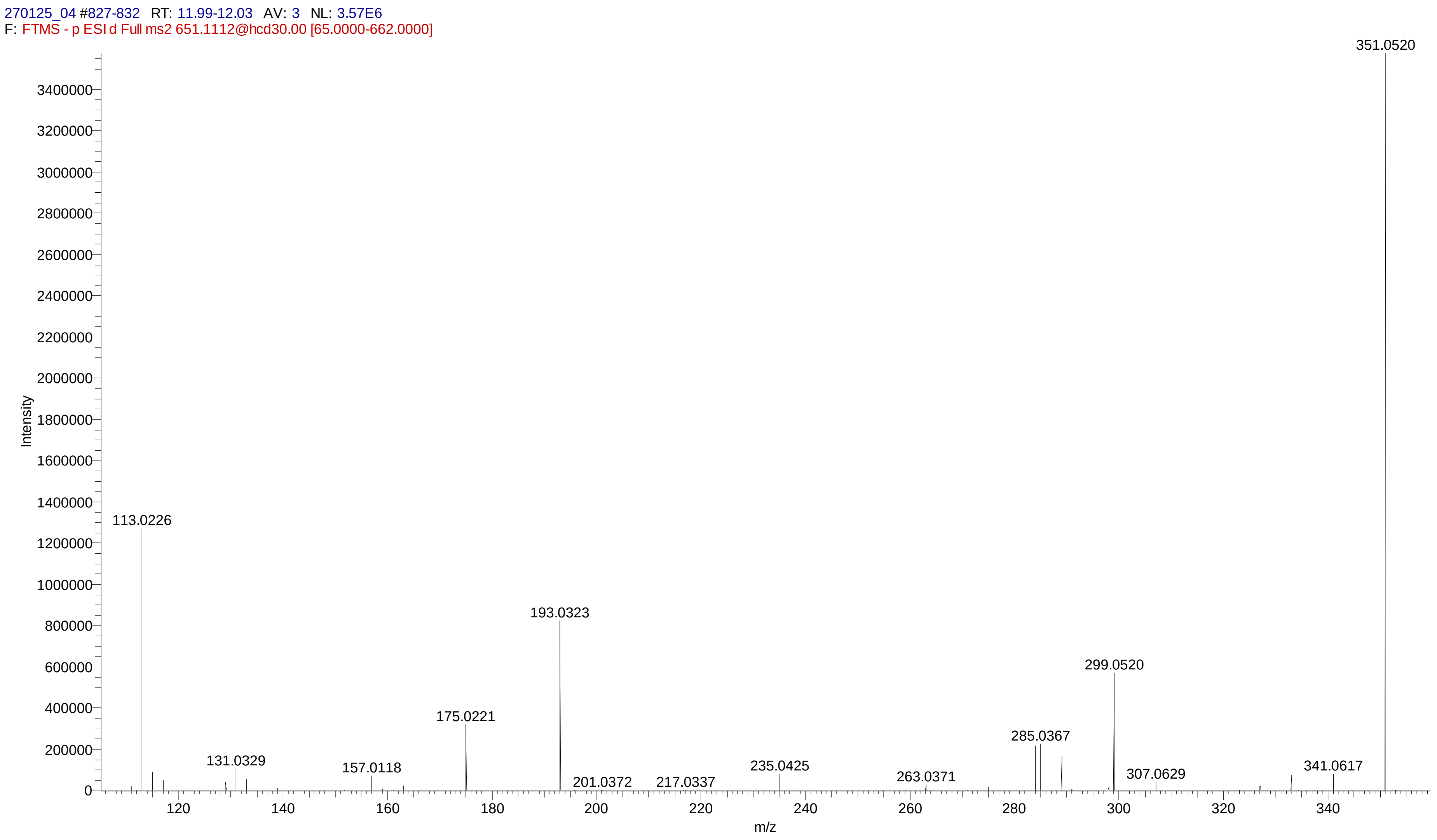

Figure S13. MS/MS spectrum of compound **11** (O-(O-Glucuronyl-O-glucuronide)methoxylated flavonoid) using ESI in negative ionization mode.

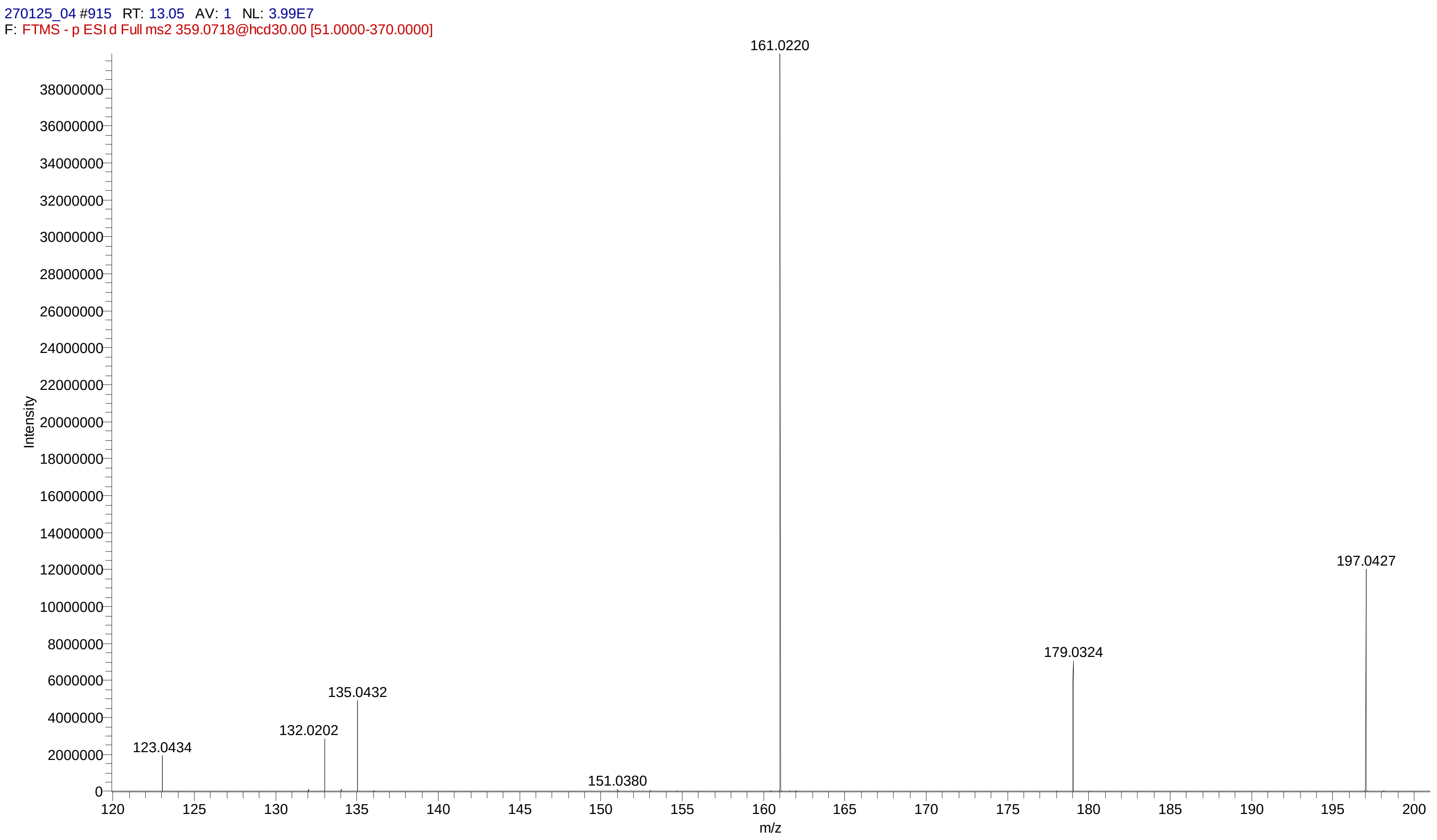

Figure S14. MS/MS spectrum of compound **12** (Rosmarinic acid) using ESI in negative ionization mode.

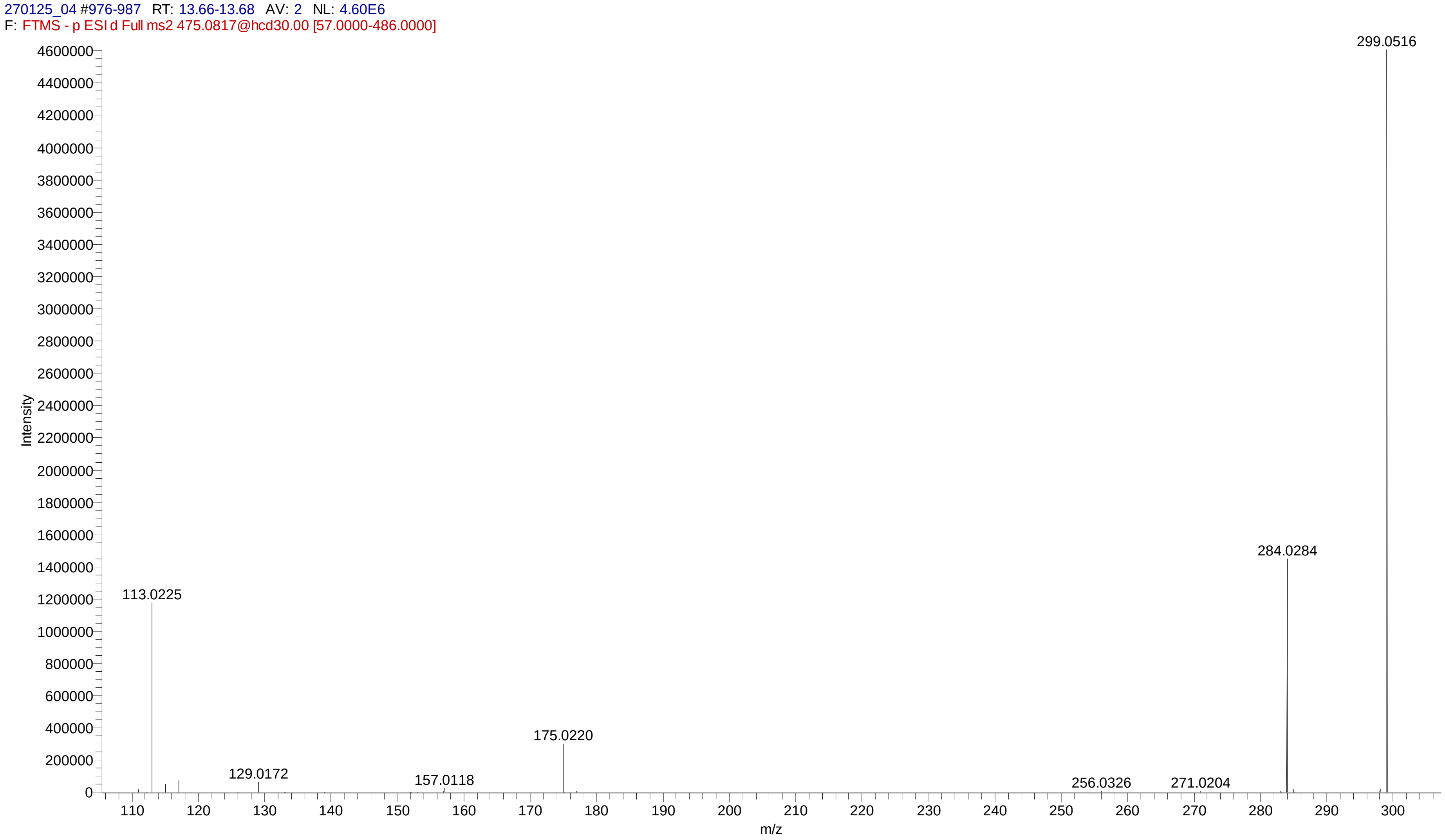

Figure S15. MS/MS spectrum of compound **13** (Methoxylated flavonoid-O-glucuronide) using ESI in negative ionization mode.

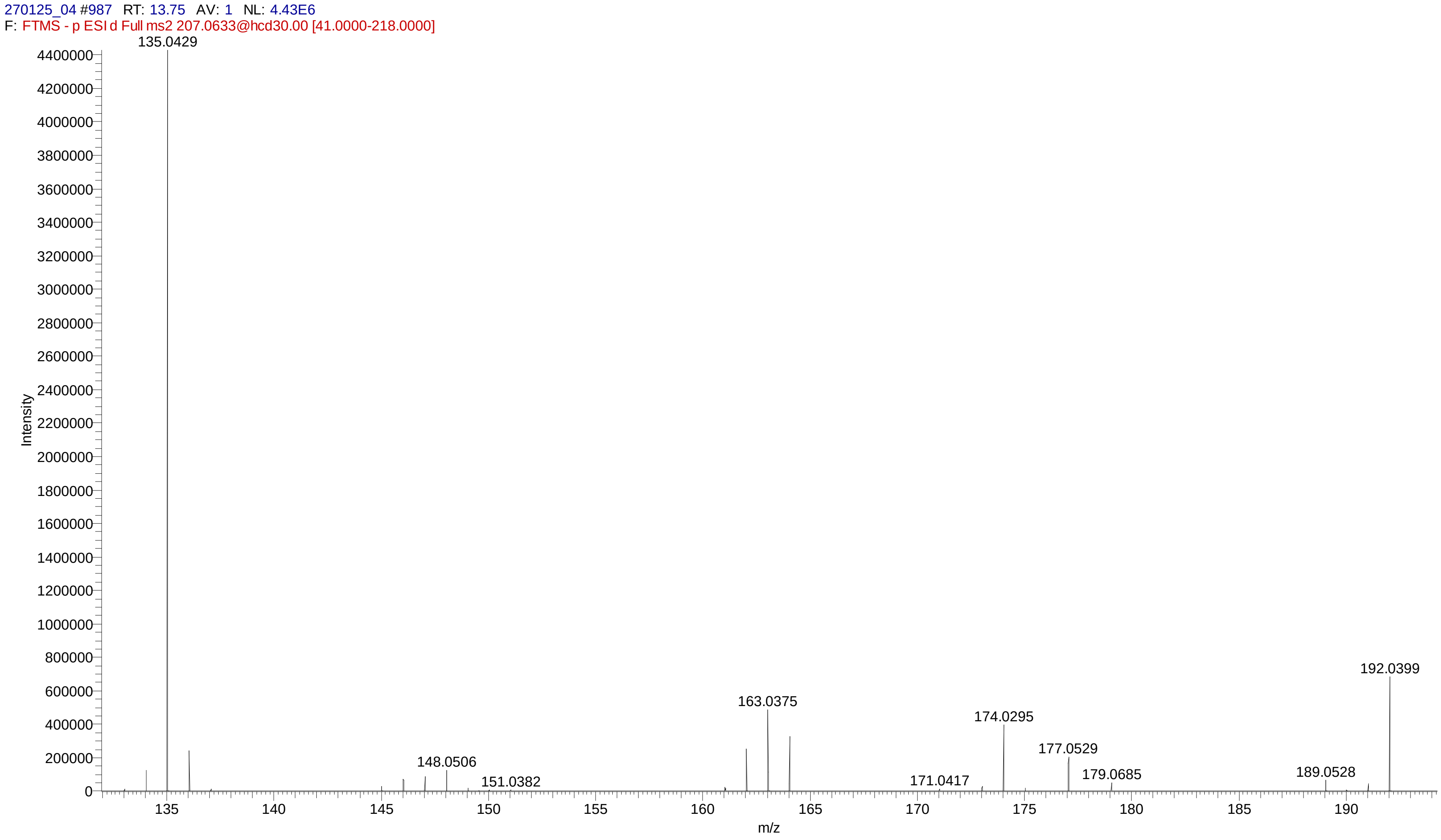

Figure S16. MS/MS spectrum of compound **14** (Unknown) using ESI in negative ionization mode.

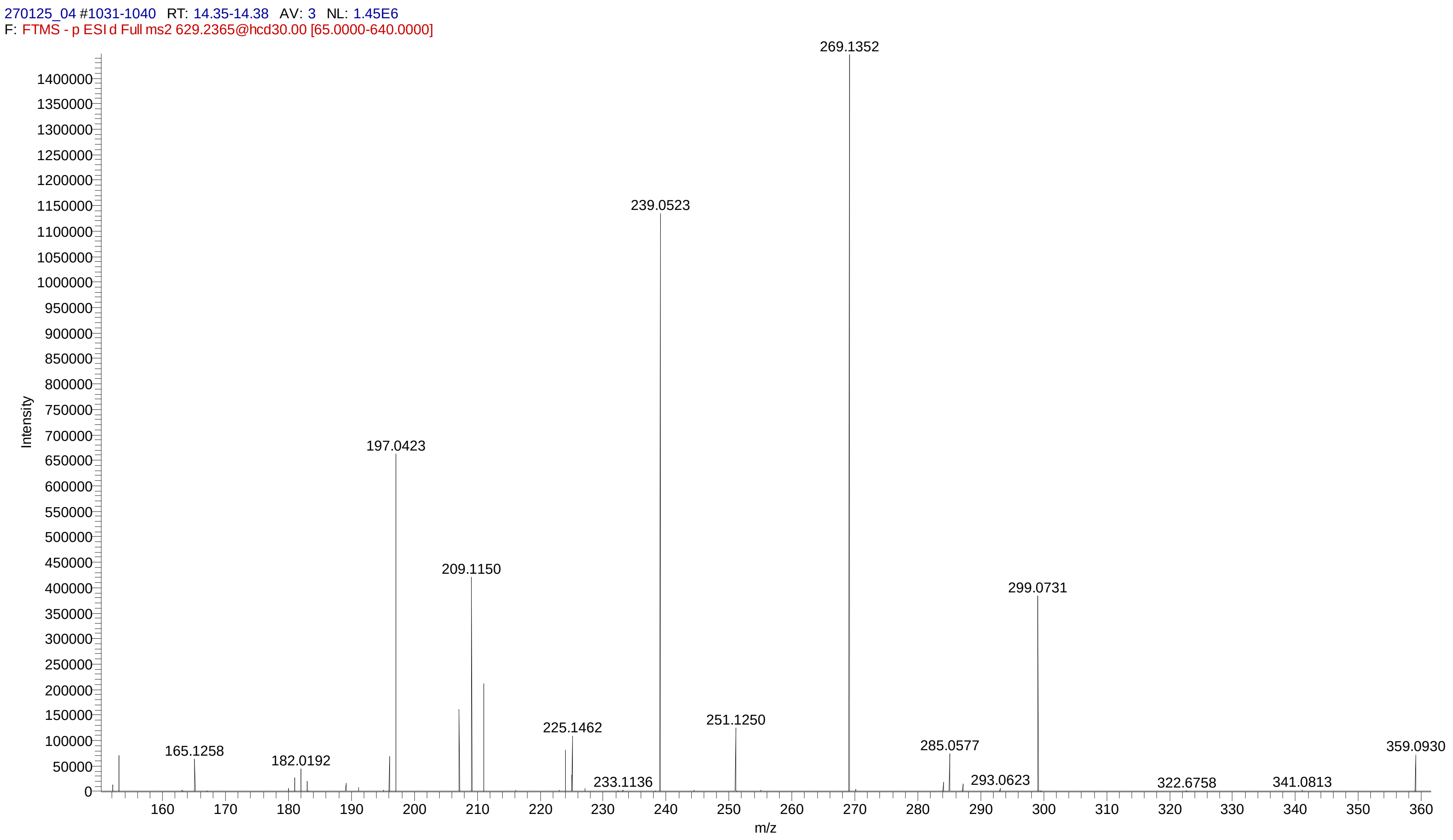

Figure S17. MS/MS spectrum of compound **15** (Unknown) using ESI in negative ionization mode.

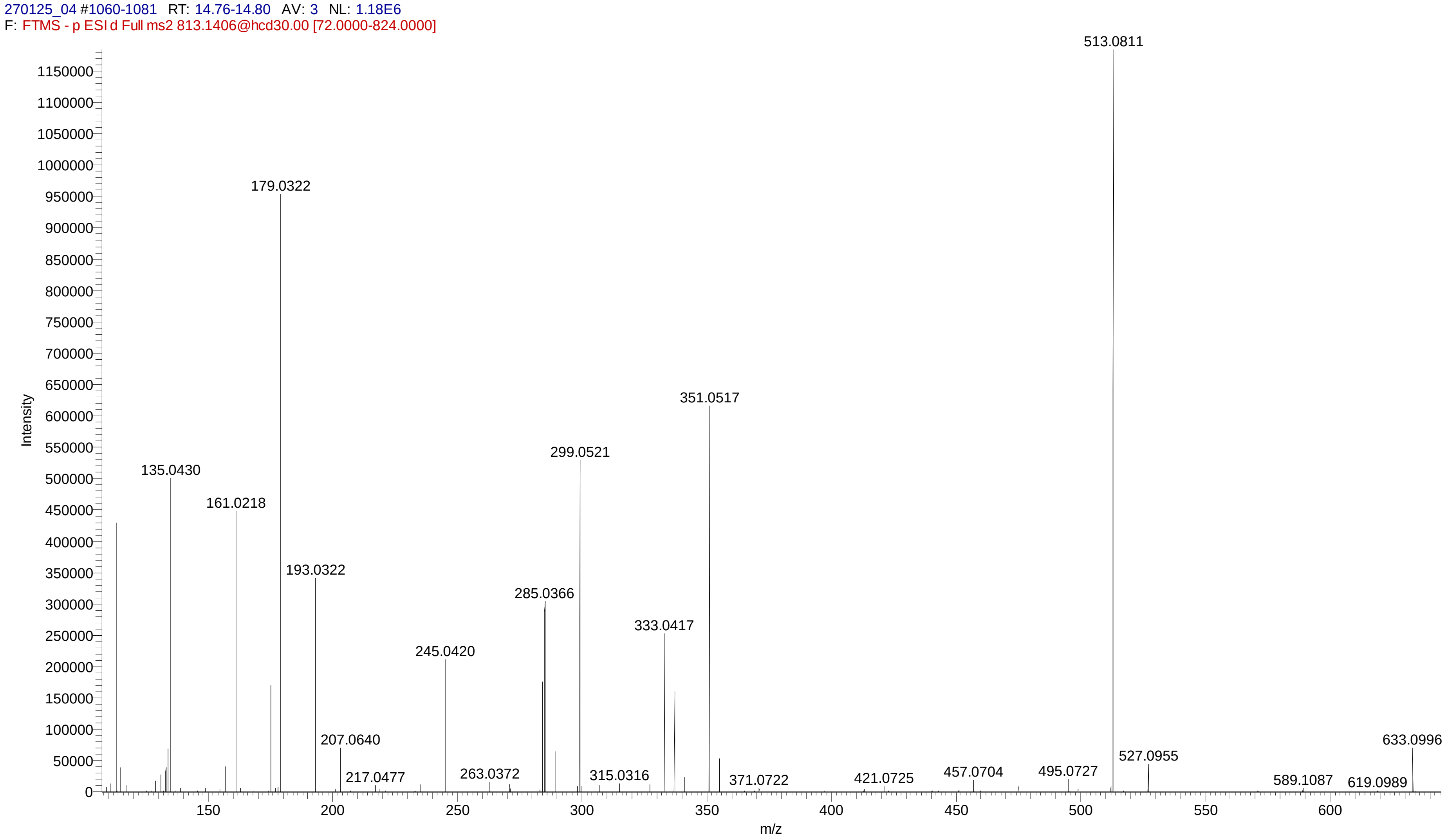

Figure S18. MS/MS spectrum of compound **16** (*O*-(*O*-Caffeoyl-*O*-glucuronyl-*O*-glucuronide)methoxylated flavonoid) using ESI in negative ionization mode.

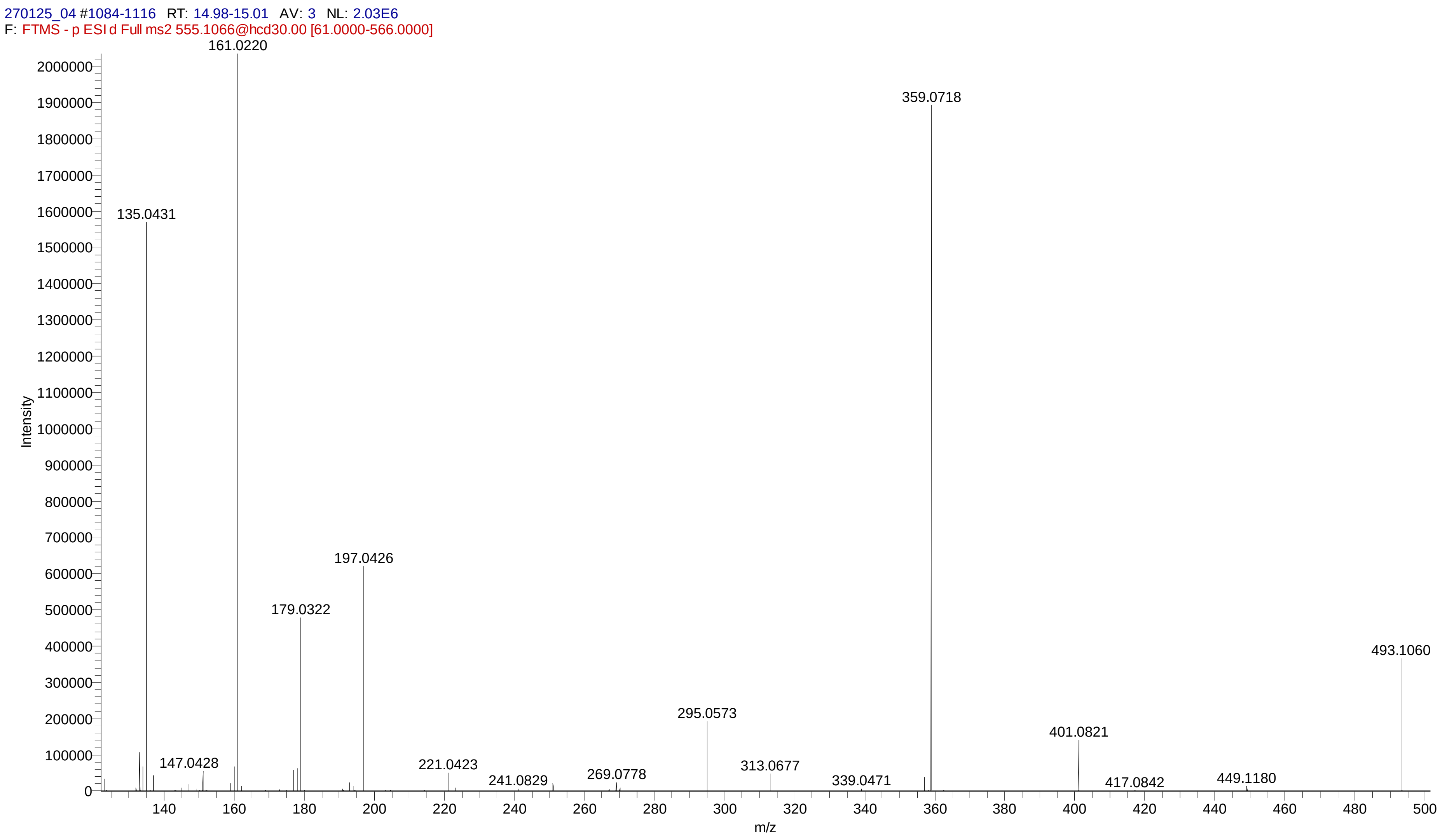

Figure S19. MS/MS spectrum of compound **17** (Salvianolic acid K) using ESI in negative ionization mode.

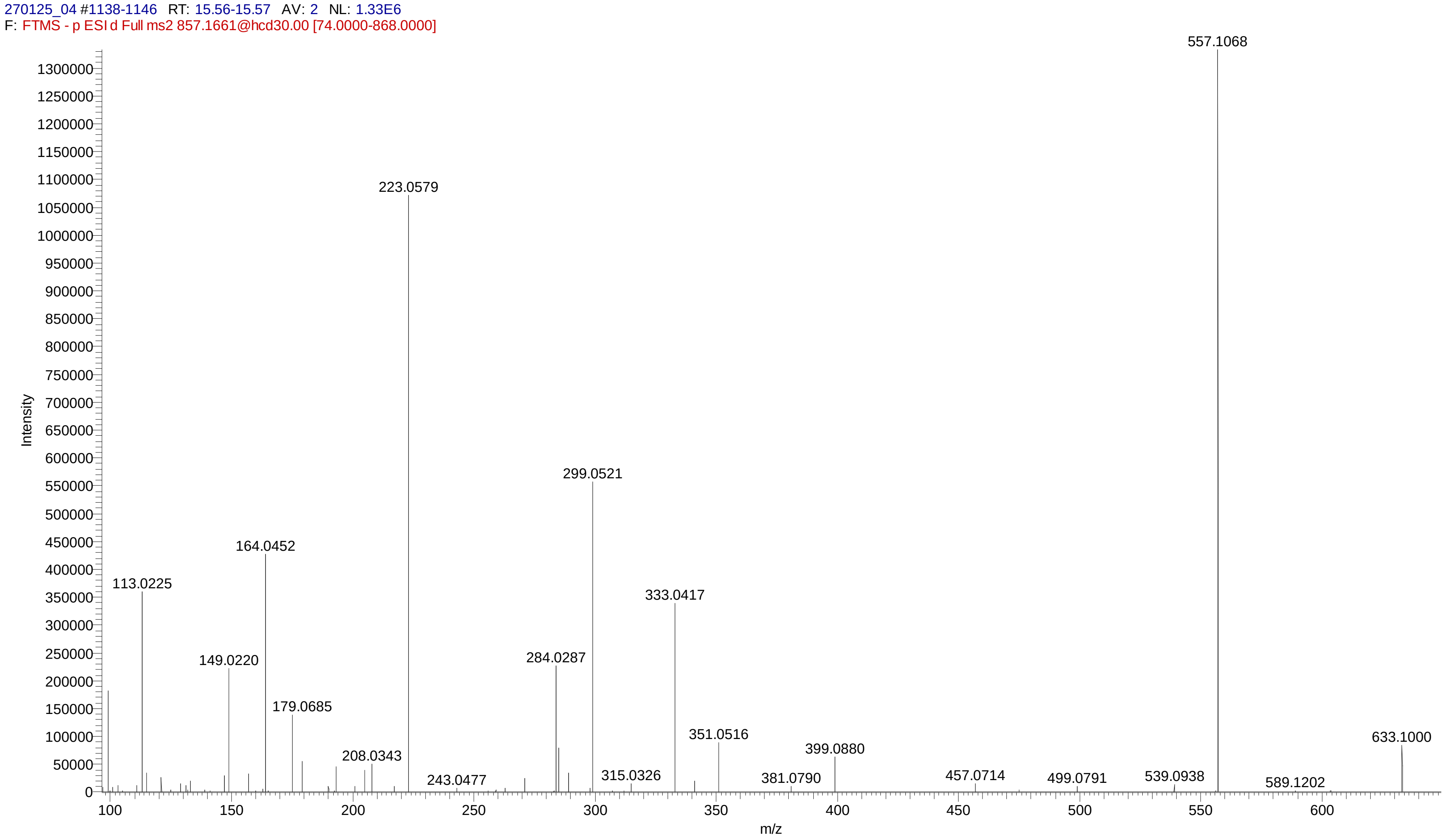

Figure S20. MS/MS spectrum of compound **18** (*O*-(*O*-Sinapoyl-*O*-glucuronyl-O-glucuronide)methoxylated flavonoid) using ESI in negative ionization mode.

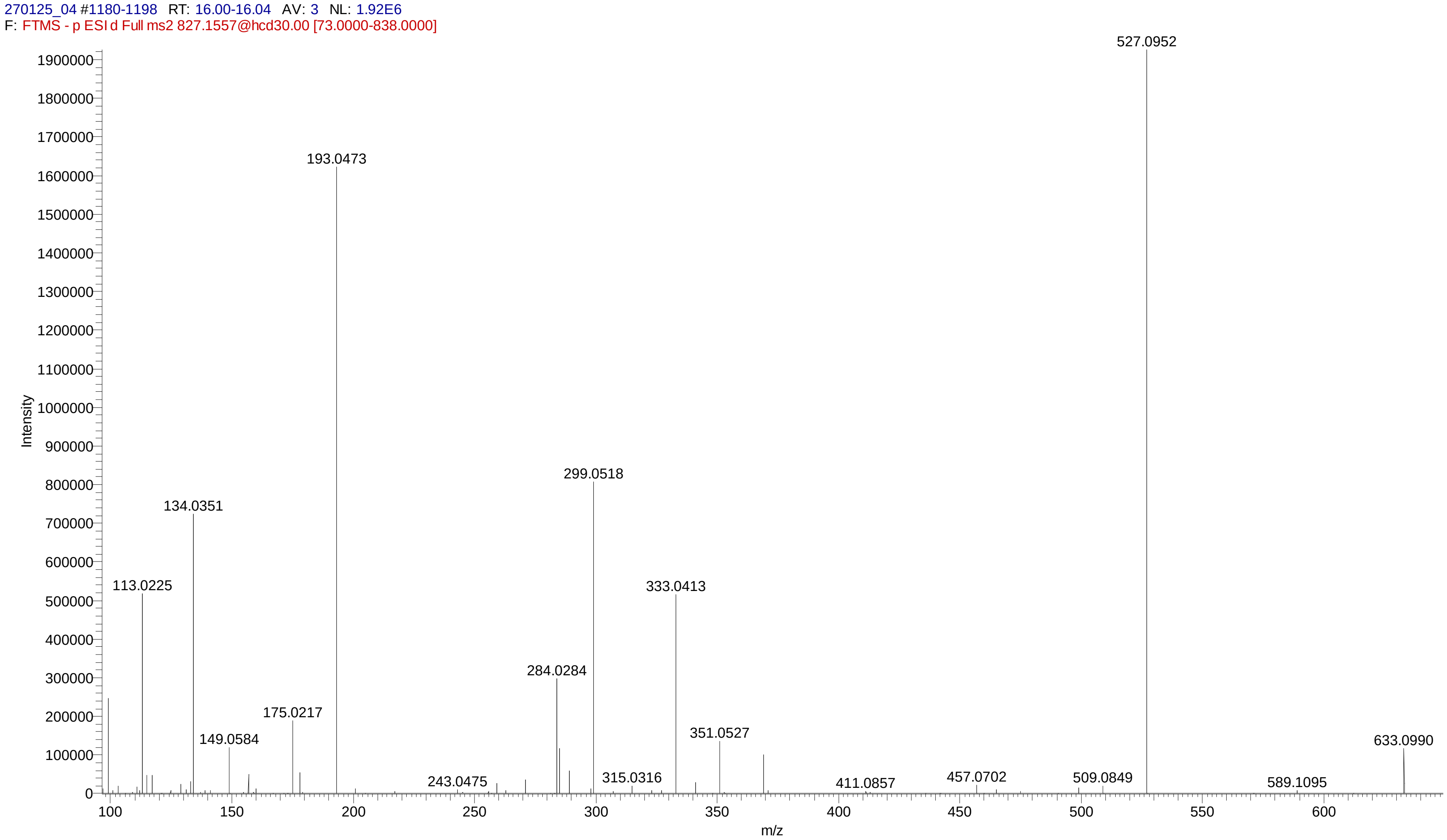

Figure S21. MS/MS spectrum of compound **19** (*O*-(*O*-Feruloyl-*O*-glucuronyl-*O*-glucuronide)methoxylated flavonoid) using ESI in negative ionization mode.

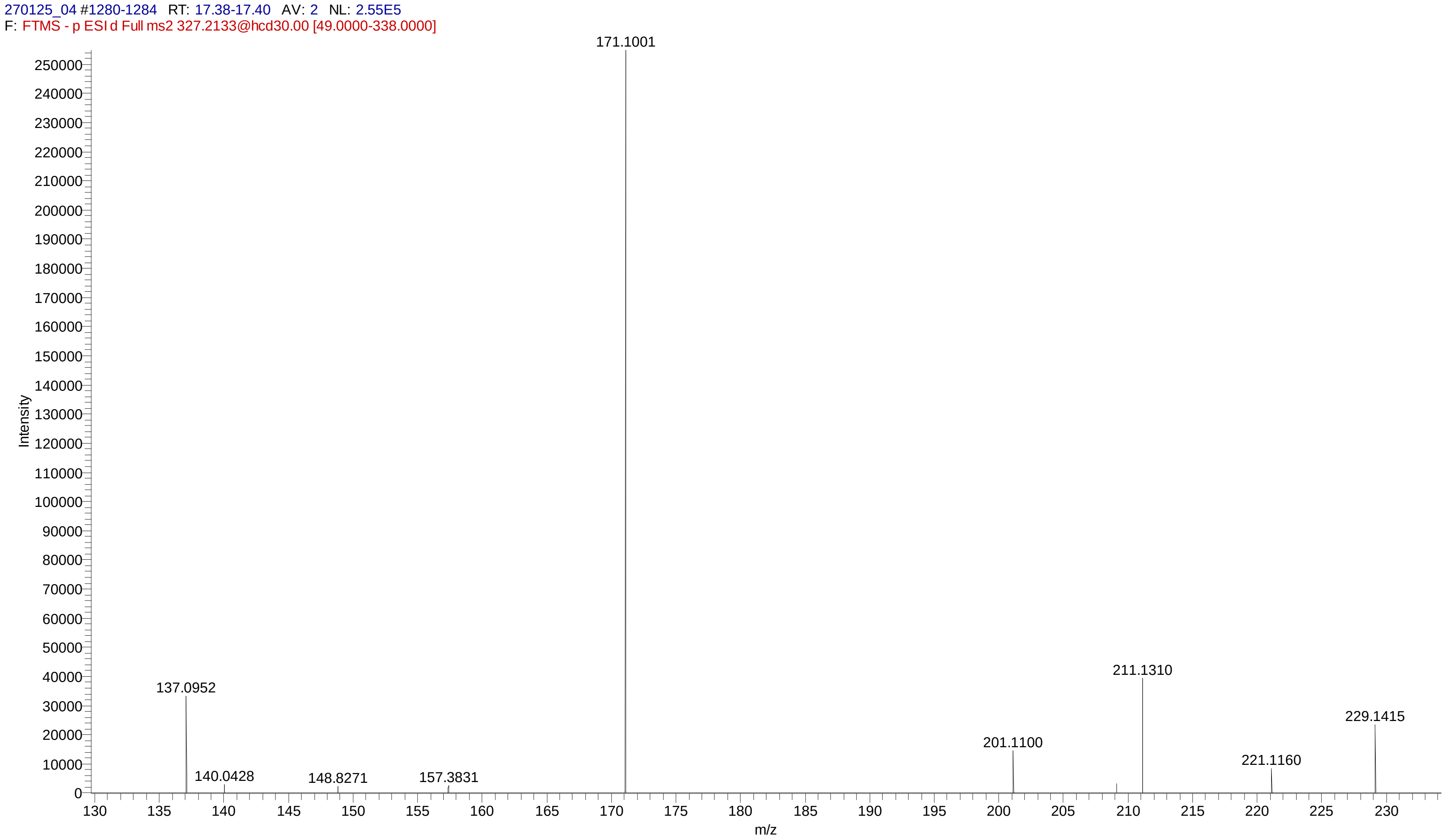

Figure S22. MS/MS spectrum of compound **20** (Dihydroxy-oxooctadecenoic acid) using ESI in negative ionization mode.

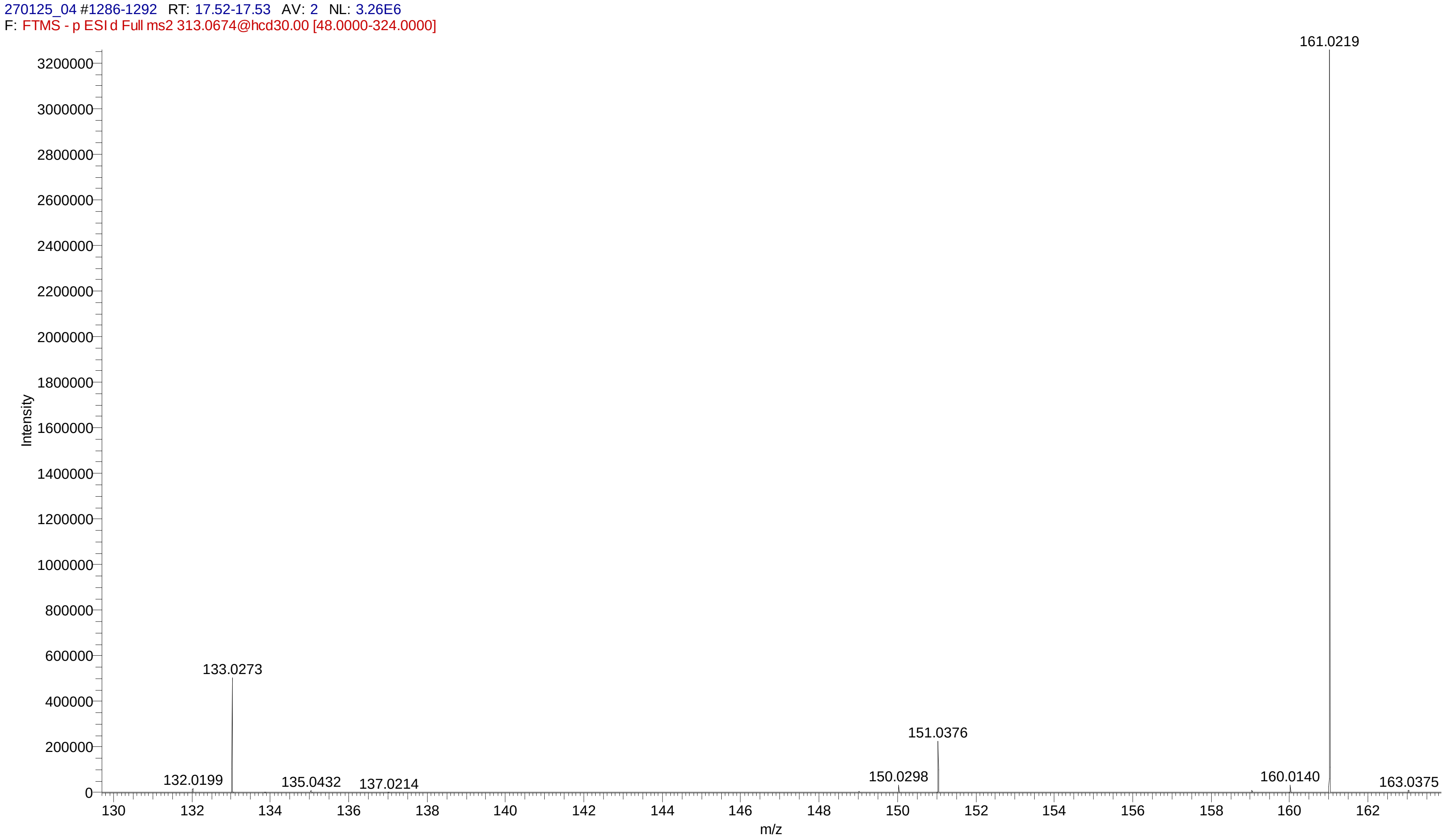

Figure S23. MS/MS spectrum of compound **21** (2-(3,4-dihydroxyphenyl)ethenyl 3-(3,4-dihydroxyphenyl)prop-2-enoate) using ESI in negative ionization mode.

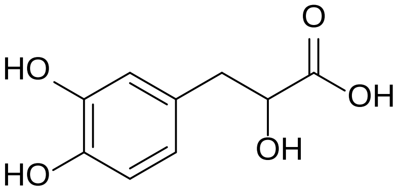

5

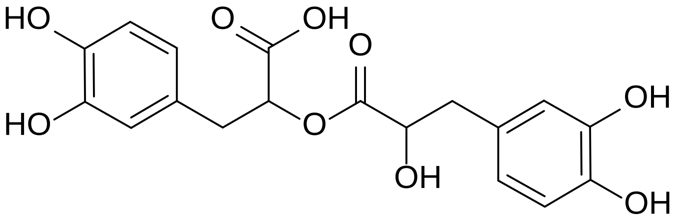

9

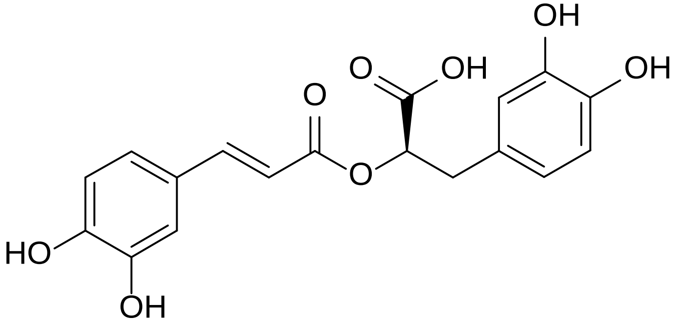

12

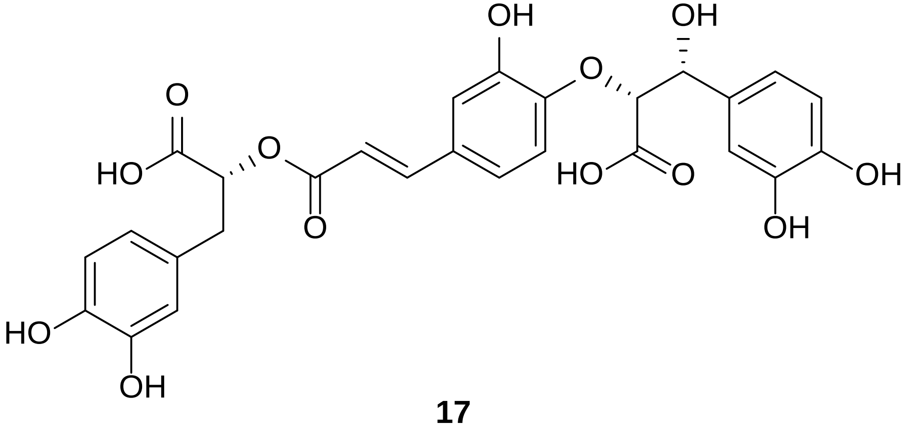

17

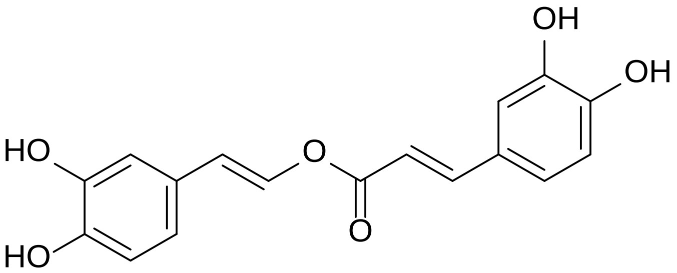

21

Figure S24. Molecular structure of several compounds of the extract of *Salvia aethiopis*.
